# Inhibition of USP14 promotes TNFα-induced cell death in head and neck squamous cell carcinoma (HNSCC)

**DOI:** 10.1038/s41418-023-01144-x

**Published:** 2023-04-13

**Authors:** Ethan L. Morgan, Tiffany Toni, Ramya Viswanathan, Yvette Robbins, Xinping Yang, Hui Cheng, Sreenivasulu Gunti, Angel Huynh, Anastasia L. Sowers, James B. Mitchell, Clint T. Allen, Zhong Chen, Carter Van Waes

**Affiliations:** 1 Tumor Biology Section, Head and Neck Surgery Branch, National Institute on Deafness and Other Communication Disorders, National Institutes of Health, Bethesda, MD USA; 2 NIH Medical Research Scholars Program, Bethesda, MD USA; 3 Translational Tumor Immunology Program, National Institute on Deafness and Other Communication Disorders, National Institutes of Health, Bethesda, MD USA; 4 Sinonasal and Skull Base Tumor Program, National Institute on Deafness and Other Communication Disorders, National Institutes of Health, Bethesda, MD USA; 5 Radiation Biology Branch, Center for Cancer Research, National Cancer Institute, National Institutes of Health, Bethesda, MD USA; 6 Present Address: School of Life Sciences, University of Sussex, Brighton, BN1 9QG UK

**Keywords:** Cancer, Deubiquitylating enzymes

## Abstract

TNFα is a key mediator of immune, chemotherapy and radiotherapy-induced cytotoxicity, but several cancers, including head and neck squamous cell carcinomas (HNSCC), display resistance to TNFα due to activation of the canonical NFκB pro-survival pathway. However, direct targeting of this pathway is associated with significant toxicity; thus, it is vital to identify novel mechanism(s) contributing to NFκB activation and TNFα resistance in cancer cells. Here, we demonstrate that the expression of proteasome-associated deubiquitinase USP14 is significantly increased in HNSCC and correlates with worse progression free survival in Human Papillomavirus (HPV)- HNSCC. Inhibition or depletion of USP14 inhibited the proliferation and survival of HNSCC cells. Further, USP14 inhibition reduced both basal and TNFα-inducible NFκB activity, NFκB-dependent gene expression and the nuclear translocation of the NFκB subunit RELA. Mechanistically, USP14 bound to both RELA and IκBα and reduced IκBα K48-ubiquitination leading to the degradation of IκBα, a critical inhibitor of the canonical NFκB pathway. Furthermore, we demonstrated that b-AP15, an inhibitor of USP14 and UCHL5, sensitized HNSCC cells to TNFα-mediated cell death, as well as radiation-induced cell death in vitro. Finally, b-AP15 delayed tumor growth and enhanced survival, both as a monotherapy and in combination with radiation, in HNSCC tumor xenograft models in vivo, which could be significantly attenuated by TNFα depletion. These data offer new insights into the activation of NFκB signaling in HNSCC and demonstrate that small molecule inhibitors targeting the ubiquitin pathway warrant further investigation as a novel therapeutic avenue to sensitize these cancers to TNFα- and radiation-induced cytotoxicity.

## Introduction

Head and neck squamous cell carcinoma (HNSCC) is the sixth most common cancer worldwide, with over 600,000 cases and 300,000 deaths annually [1]. The primary causes include alcohol consumption and smoking, with an increased number attributed to human papillomavirus (HPV) infection. The current standard therapeutic options include surgery, radiotherapy, and chemotherapy [2]. High cure rates are observed with these standard therapies in HPV + HNSCC, with a 2-year recurrence-free survival (RFS) of ≥80%. However, RFS in HPV − HNSCC remains poor, with 50% of patients experiencing tumor recurrence within 2 years. Although blockade of the programmed death (PD1) immune checkpoint pathway has been approved for patients with relapsed or metastatic HNSCC, response rates to immunotherapy are low and overall survival is poor, with a median of less than one year [3, 4]. Thus, despite advances and differences in responses to standard treatment, there is still a need for the development of better therapeutics for both HPV + and HPV- HNSCC.

TNFα is an inflammatory cytokine and a critical mediator of the cytotoxic and clinical activities of immune and radiation therapies [5, 6]. TNFα can induce cell death via the TNF receptor (TNFR) through extrinsic caspase- or necroptosis-dependent, or intrinsic reactive oxygen species and DNA damage-dependent mechanisms [7]. However, the cytotoxic effects of TNFα are often circumvented via activation of the NFκB signaling pathway, which is a critical regulator of survival and therapeutic resistance in several cancers [8–10]. We and others have shown that canonical NFκB signaling is often aberrantly activated and promotes resistance to TNFα, immune-mediated and radiotherapy-induced cytotoxicity in HNSCC [11–14]. Furthermore, The Cancer Genome Atlas (TCGA) identified that HNSCC subsets harbor genomic alterations which can modulate activation of the NFκB pathway, such as gain or activating mutations of *PIK3CA* prevalent in HPV-, or loss of *TRAF3* or gain of *HRAS* and loss of *CASP8*, which are distinct to HPV+ HNSCC [15, 16]. In addition, HPV itself induces NFκB activity via the E6 oncogene [17]. However, the underlying mechanism(s) leading to NFκB activation and TNFα resistance in HNSCC are not completely understood.

In cancer cells, the combination of genetic mutations, aneuploidy, and a high proliferative index increases protein synthesis, protein turnover, and endoplasmic reticulum (ER) stress. Cancer cells therefore display greater dependence on mechanisms that maintain protein homeostasis, including the ubiquitin-proteasome system (UPS; [18]). Protein ubiquitination is a post-translational modification essential for the regulation of cellular homeostasis [18, 19]. It is highly dynamic; ubiquitin is conjugated to protein substrates by E3 ligases and deubiquitinases readily cleave ubiquitin chains to maintain the free ubiquitin pool [20, 21].

A core component of the UPS is the 26S proteasome, a large molecular machine comprised of the proteolytic 20S core, which is capped at either end by 19S regulatory particles (RP; [22]). Ubiquitinated substrates are first recognized by the 19S RP and then translocated through a channel into the 20S core [23, 24]. The deubiquitinases Ubiquitin Specific Protease 14 (USP14) and Ubiquitin C-terminal Hydrolase L5 (UCHL5) transiently interact with the 19S RP and have pleiotropic roles in the regulation of proteasome function. These enzymes can deubiquitinate substrates at the proteasome. Prior to insertion of the substrate into the catalytic core, deubiquitination can prevent substrate degradation; however, if the substrate enters the catalytic core, deubiquitination can promote substrate degradation and ensures maintenance of the cellular ubiquitin pool [25–29]. The proteasome regulates protein degradation, preventing the toxic build-up of proteins that can occur due to higher rates of protein synthesis and ER stress in cancer cells [30, 31]. Additionally, the proteasome plays an important role in the regulation of signaling pathways; for example, degradation of IκBα is an essential step in the activation of the canonical NFκB pathway [32, 33]. Our previous pre-clinical and clinical studies with a first-generation proteasome inhibitor, Bortezomib, demonstrated inhibition of NFκB activity and anti-tumor activity in HNSCC, but these responses were transient and sustained treatment was limited by toxicity [34, 35]. Furthermore, despite the successful use of Bortezomib in cancers such as Multiple Myeloma, intrinsic resistance and tumor recurrence is a common event [36]. Thus, the targeting of other components of the UPS offer an attractive alternative to proteasome inhibition. Recent studies have revealed that the targeting of USP14 and UCHL5 with the small molecule b-AP15. can overcome proteasome inhibitor resistance in several cancers ([37]).

Here, we demonstrate that the expression of proteasomal deubiquitinase USP14 is increased in HNSCC, and the USP14/UCHL5 inhibitor b-AP15 inhibits the proliferation and survival of HNSCC cells, both in vitro and in vivo. USP14 inhibition by b-AP15 or siRNA depletion inhibited NFκB activity by preventing IκBα degradation and RELA nuclear translocation. Furthermore, we demonstrate that b-AP15 sensitizes HNSCC cells to TNFα-mediated cell death, as well as radiation-induced cell death. Thus, inhibition of specific proteasomal deubiquitinases deregulated in HNSCC warrants further investigation as a novel therapeutic avenue to sensitize these cancers to TNFα- and radiation-induced cytotoxicity.

## Results

### Proteasome-associated deubiquitinases are highly expressed in HNSCC

**Fig. 1:**
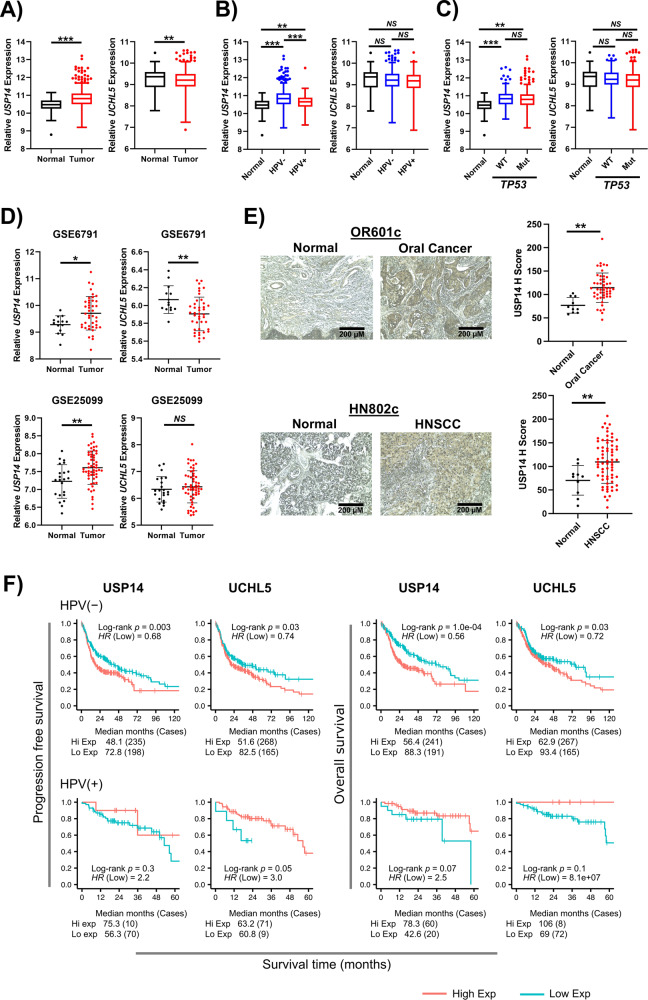
USP14 is highly expressed in HNSCC and correlates with worse progression free survival. **A** Box plot analysis of *USP14* and *UCHL5* mRNA expression in normal (*n* = 44) and HNSCC (*n* = 516) tissue from TCGA HNSCC database. **B** Box plot analysis of *USP14* and *UCHL5* mRNA expression in normal (*n* = 44), HPV- HNSCC (*n* = 434) and HPV + HNSCC (*n* = 80) tissue from TCGA HNSCC database. **C** Box plot analysis of *USP14* and *UCHL5* mRNA expression in normal (*n* = 44), WT TP53 HNSCC (*n* = 167) and mtTP53 HNSCC (*n* = 347) tissue from TCGA HNSCC database. **D** Scatter dot plot of *USP14* and *UCHL5* mRNA expression in normal and HNSCC tissue from the GEO database entries GSE6791 (normal *n* = 14, HNSCC *n* = 42) and GSE25099 (normal *n* = 22, HNSCC *n* = 57). **E** Representative immunohistochemical (IHC) staining of USP14 expression in normal and cancer tissue from OR601c and HN802c tissue microarrays (TMA). Scatter dot plot analysis of USP14 expression from the full TMAs are shown on the right. OR601c contains 10 normal and 50 oral cancer sections and HN802c contains 10 normal and 70 head and neck cancer sections. Scale bars, 200 μm. **F** Progression free and overall survival analysis of TCGA HNSCC data based on *USP14* and *UCHL5* expression, separated by HPV status. Survival data were plotted using the Kaplan–Meier survival curve. Red indicates high expression, cyan indicates low expression. *P* values were determined using the log-rank test. Plots were truncated at 10 years for HPV- patients and five years for HPV + patients, but the analyses were conducted using all data. NS not significant; *p  <  0.05; **p  <  0.01; ***p  <  0.001 (Student’s *t* test).

To identify ubiquitin-related genes involved in HNSCC, we first examined the expression of the proteasome associated deubiquitinases USP14 and UCHL5. Analysis of the TCGA HNSCC cohort demonstrated that *USP14* was expressed at significantly higher levels in HNSCC tumors, particularly HPV- tumors, than in normal tissue (Fig. 1A–C). In contrast, *UCHL5* was expressed at significantly lower levels in in HNSCC, with no difference observed based on HPV status or *TP53* mutational status (Fig. 1A–C). Similar expression trends were observed in two other cohorts of HNSCC patients (Fig. 1D). In confirmation of the transcriptomic results, we observed increased USP14 protein expression in cancer tissue from both the oral cavity and other subsides of the head and neck when compared to normal oral tissue (Fig. 1E). Finally, using TCGA cohort, we investigated the relationship between *USP14* and *UCHL5* expression and patient survival. For both *USP14* and *UCHL5*, high expression was significantly associated with worse progression free survival (PFS) and overall survival (OS) in HPV − HNSCC, but better PFS and OS in HPV + HNSCC (Fig. 1F). Taken together, these results suggest that increased expression of the proteasomal deubiquitinase USP14 may be of biological and clinical importance in HNSCC.

### The USP14 and UCHL5 inhibitor b-AP15 demonstrates anti-proliferative and pro-apoptotic activity in HNSCC cells

**Fig. 2:**
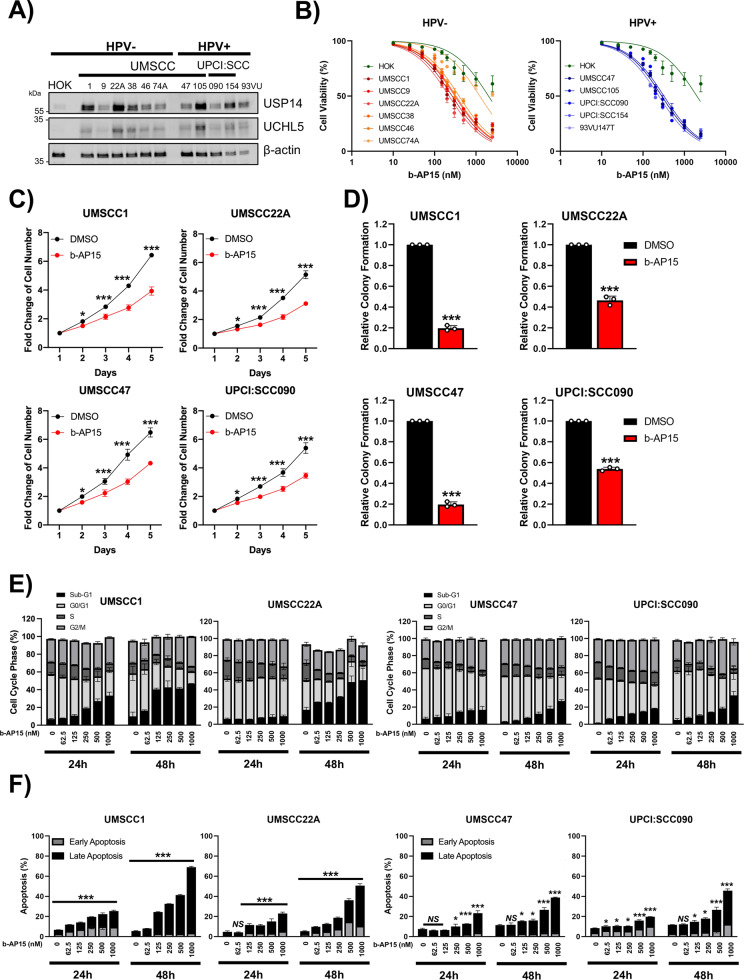
The proteasomal deubiquitinase inhibitor b-AP15 reduced proliferation and induced apoptosis in HNSCC cells. **A** Western blot analysis of USP14 and UCHL5 expression in human oral keratinocytes (HOK) and a panel of HPV- and HPV + HNSCC cell lines. β-actin was used as the loading control. **B** XTT cell viability analysis of HOK and HNSCC cell lines after treatment with increasing doses of b-AP15 (250 nM) for 48 h. **C** Cell growth analysis of UMSCC1, UMSCC22A, UMSCC47 and UPCI:SCC090 cells. Cells were treated with b-AP15 (250 nM) for 24 h. Cells were then replated at a specific cell number (cell line dependent) and counted every 24 h. **D** Colony formation assay of UMSCC1, UMSCC22A, UMSCC47 and UPCI:SCC090 cells 24 h after b-AP15 (250 nM) treatment. **E** Cell cycle analysis of UMSCC1, UMSCC22A, UMSCC47 and UPCI:SCC090 cells 24 and 48 h after treatment with increasing doses of b-AP15. **F** Annexin V analysis of UMSCC1, UMSCC22A, UMSCC47 and UPCI:SCC090 cells 24 and 48 h after treatment with increasing doses of b-AP15. Bars represent the means ± standard deviation. All experiments are representative of at least three biological replicates. NS not significant; *p  <  0.05; **p  <  0.01; ***p  <  0.001 (Student’s *t* test).

Next, we examined the expression of USP14 and UCHL5 in a panel of HNSCC cell lines previously characterised (Supplementary Table 1) [38]. Compared to human oral keratinocytes (HOKs), USP14 was expressed at higher levels in HNSCC cells, with no obvious difference between HPV + and HPV- HNSCC cells (Fig. 2A) In contrast to the data in Fig. 1, UCHL5 was also expressed at high levels when compared to HOKs. To investigate the role of proteasomal deubiquitinases in HNSCC, we studied the effects of the small molecule b-AP15, which inhibits both USP14 and UCHL5 [39]. b-AP15 treatment resulted in a dose-dependent increase in polyubiquitination as expected (Supplementary Fig. 1A). Most HPV + and HPV- HNSCC cell lines demonstrated reduced viability upon exposure to b-AP15. Only UMSCC74A cells that express wild type (WT) *TP53* [40] were insensitive to b-AP15 (Fig. 2B; IC50 values in Supplementary Table 2), with viability similar to that observed in HOK cells (IC50 > 2000 nM). b-AP15 significantly reduced the growth and colony formation capacity of HPV- (UMSCC1 and 22A) and HPV + (UMSCC47 and UPCI:SCC090) cells (Fig. 2C, D; representative images in Supplementary Fig. 2A). Similar results were observed in the full panel of HNSCC cell lines (Supplementary Fig. 3A, B). Similar to our viability data, the growth of HOK cells was not affected by b-AP15 treatment, even at doses 10 times higher than the dose used on HNSCC cells (2500 nM; Supplementary Fig. 3D).

b-AP15 treatment also altered the cell cycle, reducing cells in S phase and increasing cells in the G2/M phase in a dose dependant manner at 24 h. (Fig. 2E; representative images in Supplementary Fig. 2B). After 48 h, there was a significant increase in cells containing sub-G1 fragmented DNA, an indicator of cell death. Using an Annexin V assay, we confirmed that b-AP15 induced apoptosis in HNSCC cells (Fig. 2F and Supplementary Fig. 1C; representative images in Supplementary Fig. 4A). Mechanistically, western blot analysis demonstrated that b-AP15 induced a dose-dependent increase in cleaved caspase 3 and cleavage of its substrate PARP1 (Supplementary Fig. 1A). Treatment with the pan-caspase inhibitor Z-VAD-FMK and/or the RIP kinase inhibitor Necrostatin-1 confirmed that b-AP15-induced both caspase and RIP kinase dependent cell death, respectively (Supplementary Fig. 1B, C). Together, these data demonstrate that inhibition of proteasomal deubiquitinases inhibits the proliferation and survival of HNSCC cells.

### USP14 inhibition is the primary mediator of b-AP15-induced cytotoxicity in HNSCC cells

**Fig. 3:**
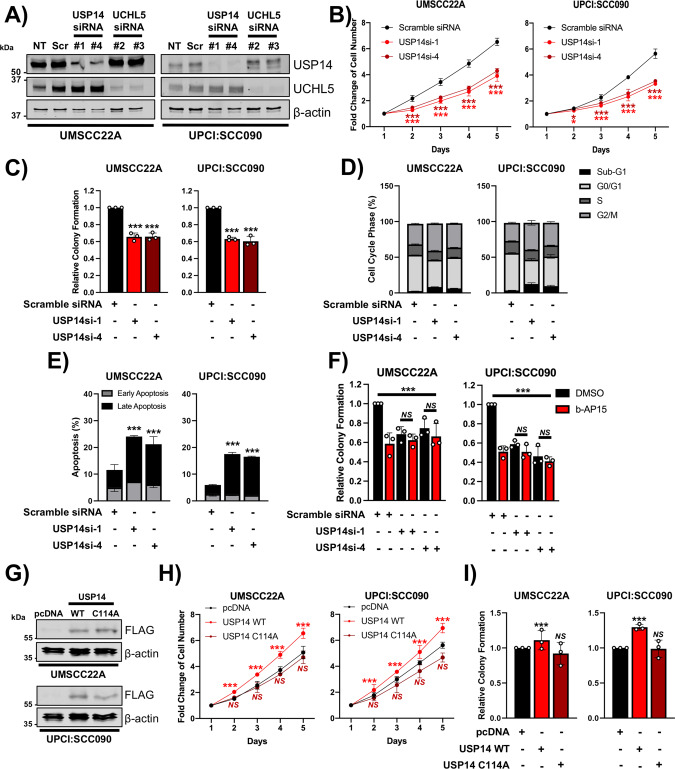
USP14 is the primary target of b-AP15 in HNSCC cells. **A** Representative western blot of USP14 and UCHL5 expression in UMSCC22A and UPCI:SCC090 cells after transfection with two specific USP14 or UCHL5 siRNAs for 72 h. β-actin was used as the loading control. **B** Cell growth analysis of UMSCC22A and UPCI:SCC090 cells after transfection of two specific USP14 siRNAs for 72 h. **C** Colony formation assay of UMSCC22A and UPCI:SCC090 cells after transfection with two specific USP14 or UCHL5 siRNA for 72 h. **D** Cell cycle analysis of UMSCC22A and UPCI:SCC090 cells after transfection with two specific USP14 siRNAs for 72 h. **E** Annexin V analysis of UMSCC22A and UPCI:SCC090 cells transfection with two specific USP14 siRNAs for 72 h. **F** Colony formation assay of UMSCC22A and UPCI:SCC090 cells transfection with two specific USP14 siRNAs for 72 h. After 48 h, cells were additionally treated with b-AP15 (250 nM) or vehicle control. **G** Representative western blot of USP14 WT and USP14 C11A expression in UMSCC22A and UPCI:SCC090 cells 48 h after transfection. β-actin was used as the loading control. **H** Cell growth analysis of UMSCC22A and UPCI:SCC090 cells after transfection with USP14 WT and USP14 C11A for 48 h. **I** Colony formation assay of UMSCC22A and UPCI:SCC090 cells after transfection with USP14 WT and USP14 C11A expression for 48 h. Bars represent the means ± standard deviation. All experiments are representative of at least three biological replicates. NS not significant; *p  <  0.05; **p  <  0.01; ***p  <  0.001 (Student’s *t* test).

b-AP15 is an inhibitor of both USP14 and UCHL5. To determine the relative contribution of these genes to HNSCC cell growth and survival, we individually depleted both genes in UMSCC22A and UPCI:SCC090 cells by siRNA knockdown (Fig. 3A). Depletion of USP14 significantly decreased cell growth and colony formation, whereas UCHL5 depletion had minimal impact (Fig. 3B, C, Supplementary Fig. 5A, B; representative images in Supplementary Figs. 6A and 7A). USP14 depletion resulted in reduced cells in S phase, an accumulation of cells in the G2/M phase and induced apoptosis; however, this was to a lesser extent than treatment with b-AP15, likely due to more selective targeting of USP14 (Fig. 3D, E; representative images in Supplementary Fig. 6B, C). Conversely, UCHL5 depletion did not significantly alter the cell cycle and induced minimal apoptosis in HNSCC cells (Supplementary Fig. 5C, D; representative images in Supplementary Fig. 7B, C). To further confirm that USP14 was the primary target of b-AP15 in HNSCC, we treated USP14 and UCHL5 depleted cells with b-AP15. In control and UCHL5 depleted cells, b-AP15 significantly reduced colony formation as previously demonstrated. However, in USP14 depleted cells, b-AP15 treatment did not further reduce colony formation, suggesting that the inhibitory effects of b-AP15 require USP14 (Fig. 3F, Supplementary Fig. 3E; representative images in Supplementary Figs. 6D and 7D).

To validate our siRNA data, we used a more specific inhibitor of USP14, IU1-47 [41]. Similar to b-AP15, exposure of HNSCC cells to IU1-47 significantly inhibited cell growth and colony formation and resulted in a dose-dependent increase in apoptosis (Supplementary Fig. 8A–C). Finally, we investigated whether exogenous expression of USP14 could enhance cell growth in HNSCC cells. Over-expression of wild type USP14 enhanced cell growth and colony formation in both UMSCC22A and UPCI:SCC090 cells (Fig. 3G–I). Importantly, the increased cell growth was dependent on the catalytic activity of USP14, as the catalytically inactive mutant USP14 C114A [42], failed to increase cell growth. Taken together, these data demonstrate that USP14 promotes the proliferation and survival of HNSCC cells in a catalytically dependent manner and is a functionally important target of b-AP15.

### USP14 inhibition regulates NFκB activity by reducing TNF-mediated IκB degradation and nuclear translocation of RELA

**Fig. 4:**
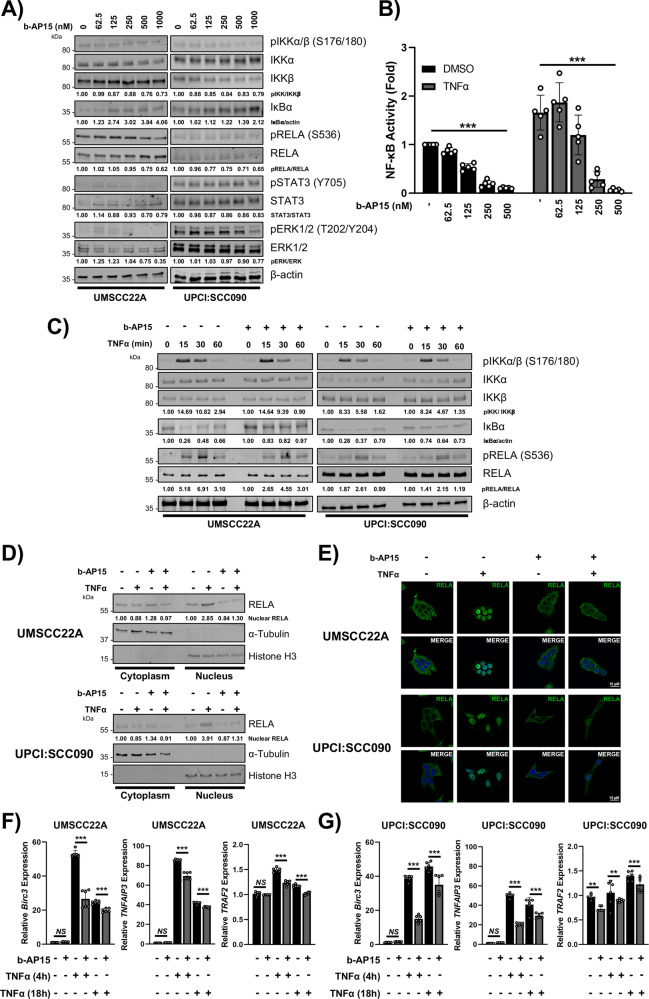
b-AP15 reduces NFκB activity by preventing the nuclear translocation of RELA. **A** Representative western blot of the expression of NFκB pathway components, STAT3 and ERK1/2 in UMSCC22A and UPCI:SCC090 after treatment with increasing doses of b-AP15 for 24 h. β-actin was used as the loading control. **B** NFκB reporter activity after treatment with increasing doses of b-AP15. Cells were treated with increasing doses of b-AP15 or vehicle control for 24 h, with TNFα (20 ng/mL) added for the final 16 h. **C** Representative western blot of the phosphorylation and expression of IKKα/β, IκBα and RELA. UMSCC22A and UPCI:SCC090 cells were treated with b-AP15 (250 nM) or vehicle control for 6 h, with TNFα (20 ng/mL) added for the specified time. β-actin was used as the loading control. **D** Representative western blot of the RELA localisation after cellular fractionation into cytoplasmic and nuclear fractions. UMSCC22A and UPCI:SCC090 cells were treated with b-AP15 (250 nM) or vehicle control for 6 h, with TNFα (20 ng/mL) added for 30 min. α-tubulin and Histone H3 were used as loading controls for the cytoplasmic and nuclear fractions, respectively. **E** Representative immunofluorescence images of RELA localisation. UMSCC22A and UPCI:SCC090 cells were treated with b-AP15 (250 nM) or vehicle control for 6 h, with TNFα (20 ng/mL) added for 30 min. DAPI was used as a nuclear counterstain. qPCR analysis of *Birc3, TNFAIP3* and *TRAF2* in UMSCC22A (**F**) and UPCI:SCC090 (**G**) cells. Cells were treated with b-AP15 (250 nM) for 24 h, with TNFα (20 ng/mL) added for the indicated times. *U6* was used as the loading control. **H** Cell growth analysis of UMSCC22A and UPCI:SCC090 cells after transfection USP14 WT and USP14 C11A expression 48 h. Bars represent the means ± standard deviation. Western blot experiments are representative of at least three biological repeats; numbers represent quantification of protein bands. Immunofluorescence images are representative from 2 biological replicates. qPCR analysis data is from 3 technical repeats of 2 independent experiments. NS not significant; *p  <  0.05; **p  <  0.01; ***p  <  0.001 (Student’s *t* test).

b-AP15 has previously been shown to inhibit several signaling pathways associated with proliferation and survival, such as IκB-NFκB, ERK/MAPK, and STAT3 [43–45]. Under basal conditions, treatment with varying concentrations of b-AP15 had minimal effect on the phosphorylation or expression of IKKα, IKKβ, RELA, ERK or STAT3, except at b-AP15 concentrations ≥500 nM (Fig. 4A), which is a higher concentration than which effects on cell cycle, apoptosis and colony formation were observed. By contrast, lower concentrations of b-AP15 (125–250 nM) enhanced basal IκB, consistent with the known role of the UPS in IκB degradation. As TNF signalling is known to promote IKK-dependent UPS-mediated IκB proteolysis, as well as phosphorylation and transactivation of RELA, cells were treated without or with TNFα after pre-treatment with b-AP15. TNFα induced peak IKK phosphorylation and IκB degradation after 15 min, and peak RELA phosphorylation after 30 min (Fig. 4C). Although low dose b-AP15 alone had no effect on TNF-induced IKKα/β phosphorylation and minimal effect on RELA phosphorylation, b-AP15 prevented TNF-induced IκBα degradation. Since IκB masks the Nuclear Localization Sequence (NLS) and DNA binding cleft of RELA that promotes transcriptional activity, we further investigated the impact of b-AP15 on NFκB activity. Increasing doses of b-AP15 significantly inhibited both basal and TNFα-induced NFκB activity even at low doses, as demonstrated using an HPV- UMSCC1 HNSCC reporter cell line previously established in our lab ([13]; Fig. 4B). Both USP14 depletion and IU1-47 exposure also reduced NFκB activity, whereas UCHL5 depletion did not (Supplementary Fig. 9A–C).

To examine further if the effects on NF-κB activity were related to IκB-dependent nuclear translocation of RELA, we assessed nuclear translocation using nuclear fractionation and immunofluorescence analysis. b-AP15 pre-treatment significantly inhibited TNFα-induced RELA nuclear translocation (Fig. 4D, E; quantification in Supplementary Fig. 9D). In addition, treatment with IU1-47 or USP14 siRNA also led to decreased TNFα-induced RELA nuclear translocation, whereas UCHL5 siRNA did not (Supplementary Fig. 9E–H). Finally, to confirm that b-AP15 inhibits NFκB activity, we analyzed the expression of the well characterised NFκB target genes *BIRC3, TNFAIP3* and *TRAF2*. b-AP15 inhibited TNFα-induced expression of all three genes, at both early and late times points (Fig. 4F, G). Taken together, these data demonstrate that USP14 activity is required for RELA nuclear translocation, NFκB reporter activity and NFκB-dependent gene expression in HNSCC cells.

### USP14 deubiquitinates IkBα and promotes its degradation

**Fig. 5:**
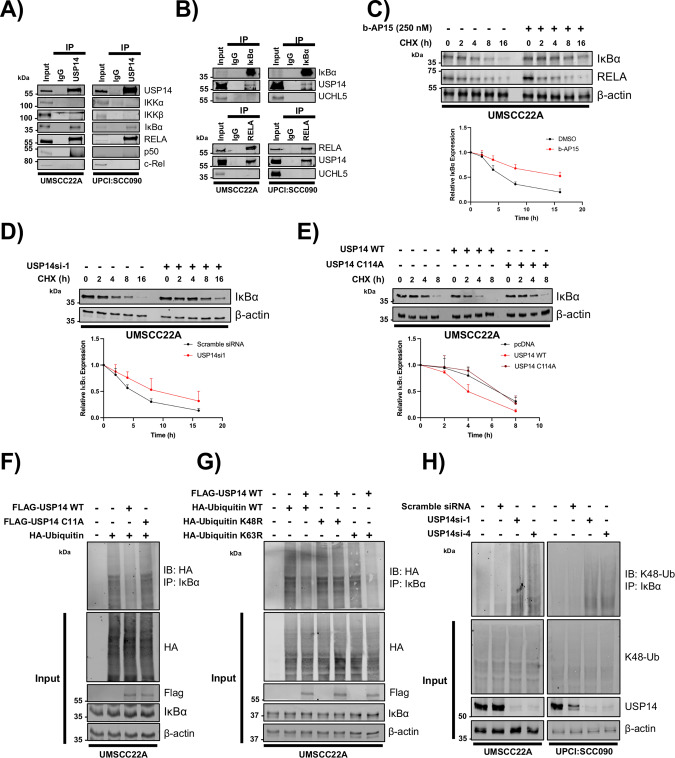
USP14 interacts with and deubiquitinates IκBα. **A** Representative western blot of endogenous USP14 immunoprecipitation in UMSCC22A and UPCI:SCC090 cells. USP14 immunoprecipitates were analyzed for binding to the indicated components of the NFκB pathway. USP14 was detected to confirm successful immunoprecipitation. **B** Representative western blot of endogenous IκBα and RELA immunoprecipitation in UMSCC22A and UPCI:SCC090 cells. IκBα and RELA immunoprecipitates were analyzed for binding to the USP14 and UCHL5. IκBα and RELA, respectively, were detected to confirm successful immunoprecipitation. **C** Representative western blot of UMSCC22A cells after treatment with b-AP15 (250 nM) for 6 h. Cells were the treated with 20 µM cycloheximide and harvested at the indicated time points. Lysates were analyzed for the expression of IκBα and RELA. β-actin was used as the loading control. Quantification of the protein band intensities from three biological repeats are shown below. **D** Representative western blot of UMSCC22A cells after transfection with a specific USP14 siRNA for 48 h. Cells were treated with 20 µM cycloheximide and harvested at the indicated time points. Lysates were analyzed for the expression of IκBα. β-actin was used as the loading control. Quantification of the protein band intensities from three biological repeats are shown below. **E** Representative western blot of HeLa cells after transfection of FLAG-USP14 WT or FLAG-USP14 C114A. Cells were treated with 20 µM cycloheximide and harvested at the indicated time points. Lysates were analyzed for the expression of IκBα. β-actin was used as the loading control. Quantification of the protein band intensities from three biological repeats are shown below. **F** UMSCC22A cells were co-transfected with HA-Ubiquitin and Flag-USP14 WT or FLAG-USP14 C114A. Cells were treated with 10 µM MG132 for 6 h and IκBα was immunoprecipitated. Ubiquitinated IκBα was detected using an anti-HA antibody. β-actin was used as the loading control. **G** UMSCC22A cells were co-transfected with HA-Ubiquitin or mutant Ubiquitin (K48R or K63R), with or without Flag-USP14 WT. Cells were treated with 10 µM MG132 for 6 h and IκBα was immunoprecipitated. Ubiquitinated IκBα was detected using an anti-HA antibody. β-actin was used as the loading control. **H** UMSCC22A and UPCI:SCC090 cells were transfected with two specific USP14 siRNAs for 72 h. Cells were treated with 10 µM MG132 for 6 h before harvesting and IκBα was immunoprecipitated Ubiquitinated IκBα was detected using an anti-K-48ubiquitin antibody. β-actin was used as the loading control. Bars represent the means ± standard deviation. All experiments are representative of at least three biological replicates. NS not significant; *p  <  0.05; **p  <  0.01; ***p  <  0.001 (Student’s *t* test).

A key step in NFκB activation is the ubiquitination and proteasomal degradation of IκBα [32, 33]. As a deubiquitinase, USP14 regulates protein expression by removing ubiquitin chains from its substrates. Therefore, we investigated whether USP14 regulates IκBα expression via its canonical deubiquitinase function. First, we performed immunoprecipitation (IP) of USP14 to identify whether any of the components of the NFκB pathway interacted with USP14. USP14 bound to both IκBα and RELA (Fig. 5A), which was confirmed by performing the reciprocal IPs (Fig. 5B). Neither RELA nor IκBα bound to UCHL5 (Fig. 5B). Using a cycloheximide chase assay, we observed that b-AP15 treatment significantly enhanced the stability of IκBα, but not RELA (Fig. 5C). USP14 depletion similarly enhanced IκBα stability, whereas WT USP14, but not the catalytic mutant, decreased IκBα stability (Fig. 5D, E).

Next, UMSCC22A cells were transfected with FLAG-USP14, FLAG-USP14 C114A and HA-Ubiquitin, and IκBα immunoprecipitates were probed for ubiquitination using the HA-antibody. USP14 significantly reduced IκBα ubiquitination, whereas the catalytic mutant had no effect (Fig. 5F). To determine which ubiquitin linkage USP14 deubiquitinates, cells were transfected with HA-tagged K48R or K63R ubiquitin mutants, which are unable to be polyubiquitinated with K48 or K63 chains, respectively. USP14 only cleaved the wild type and K63R form of ubiquitin from IκBα, but not K48R ubiquitin (Fig. 5G). These results suggest that USP14 removes K48 polyubiquitin chains from IκBα. Confirming these data, USP14 depletion resulted in enhanced K48-ubiquitinated IκBα levels (Fig. 5H). Taken together, these data demonstrate that USP14 regulates the stability of IκBα by removing K48-ubiquitin chains and promoting TNFα-induced IκBα degradation.

### USP14 inhibition enhances TNF-induced cell death

**Fig. 6:**
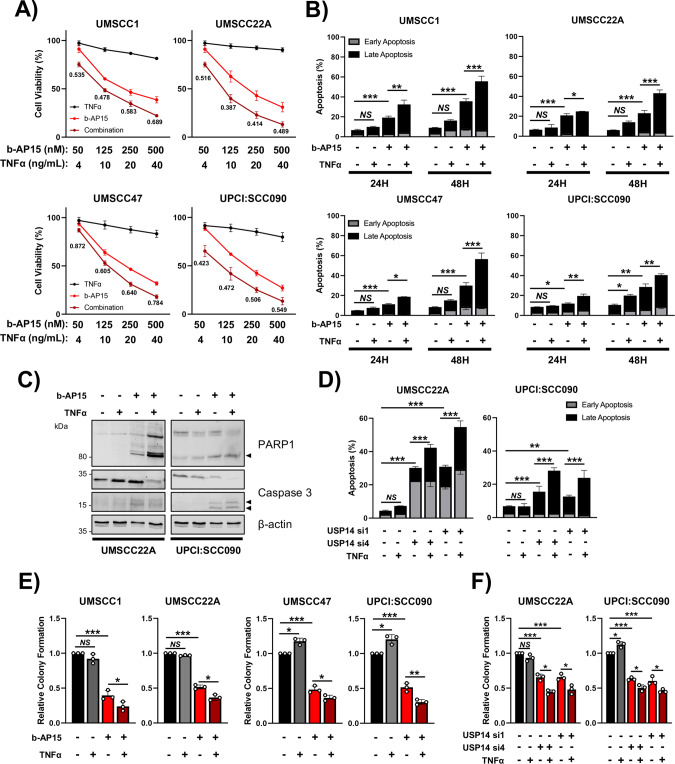
USP14 inhibition sensitizes HNSCC cells to TNFα-induced cell death. **A** XTT cell viability analysis of HNSCC cell lines after treatment with varying doses of b-AP15 and/or TNFα for 48 h. Values below the combination are Combination Indices (CI) as described in the text. **B** Annexin V analysis of HNSCC cell lines after treatment with TNFα (20 ng/mL), b-AP15 (250 nM), or the combination for 24 and 48 h. **C** Representative western blot of UMSCC22A and UPCI:SCC090 cells after treatment with TNFα (20 ng/mL), b-AP15 (250 nM) or the combination for 24 h. Lysates were analyzed for PARP1 and Caspase 3 cleavage. β-actin was used as the loading control. **D** Annexin V analysis of UMSCC22A and UPCI:SCC090 cells after transfection with two specific USP14 siRNAs for 72 h. TNFα (20 ng/mL) was added for the last 24 h. **E** Colony formation assay of HNSCC cell lines after treatment with TNFα (20 ng/mL), b-AP15 (250 nM) or the combination for 24 h. **F** Colony formation assay of UMSCC22A and UPCI:SCC090 cells after transfection with two specific USP14 siRNAs for 72 h. TNFα (20 ng/mL) was added for the last 24 h. Bars represent the means ± standard deviation. All experiments are representative of at least three biological replicates. NS not significant; *p  <  0.05; **p  <  0.01; ***p  <  0.001 (Student’s *t* test).

Canonical NFκB activity is a critical driver of TNF resistance and inhibition of the NFκB pathway can promote TNF-induced cell death [46]. Therefore, we assessed whether b-AP15 could enhance the cytotoxic effects of TNF treatment in HNSCC cells. HNSCC cells were treated with increasing does of TNF alone or in combination with b-AP15. Each cell line was relatively resistant to TNF alone (decrease in cell viability <10%); b-AP15 monotherapy significantly reduced the viability in a dose-dependent manner (Fig. 6A). The addition of exogenous TNFα to b-AP15 significantly reduced the viability in all cell lines compared with b-AP15 alone. To determine whether the decrease in viability in the combination treatment was synergistic, we calculated the combination index [47]. The CIs for these combinations were <0.9 at each dose combination, indicating a synergistic reduction in cell viability. As expected, there were minimal combinatorial effects of b-AP15 and TNFα treatment in TP53 WT cells (HOK and UMSCC74A) and this was not synergistic (CIs > 1; Supplementary Fig. 10). Combination treatment resulted in a significant increase in caspase-dependent apoptosis and inhibition of colony inhibition (Fig. 6B, C; Supplementary Fig. 11A, C). Furthermore, USP14 depletion similarly sensitized HNSCC cells to TNF-induced apoptosis (Fig. 6D; representative images in Supplementary Fig. 11D). Finally, b-AP15 treatment or USP14 depletion in combination with TNF significantly reduced colony formation compared with either treatment alone (Fig. 6E, F; representative images in Supplementary Fig. 11B, D). Altogether, these data demonstrate that therapeutic inhibition or depletion of USP14 sensitizes HNSCC cells to TNFα-induced cell death.

### b-AP15 enhances radiation-induced cell death in vitro

**Fig. 7:**
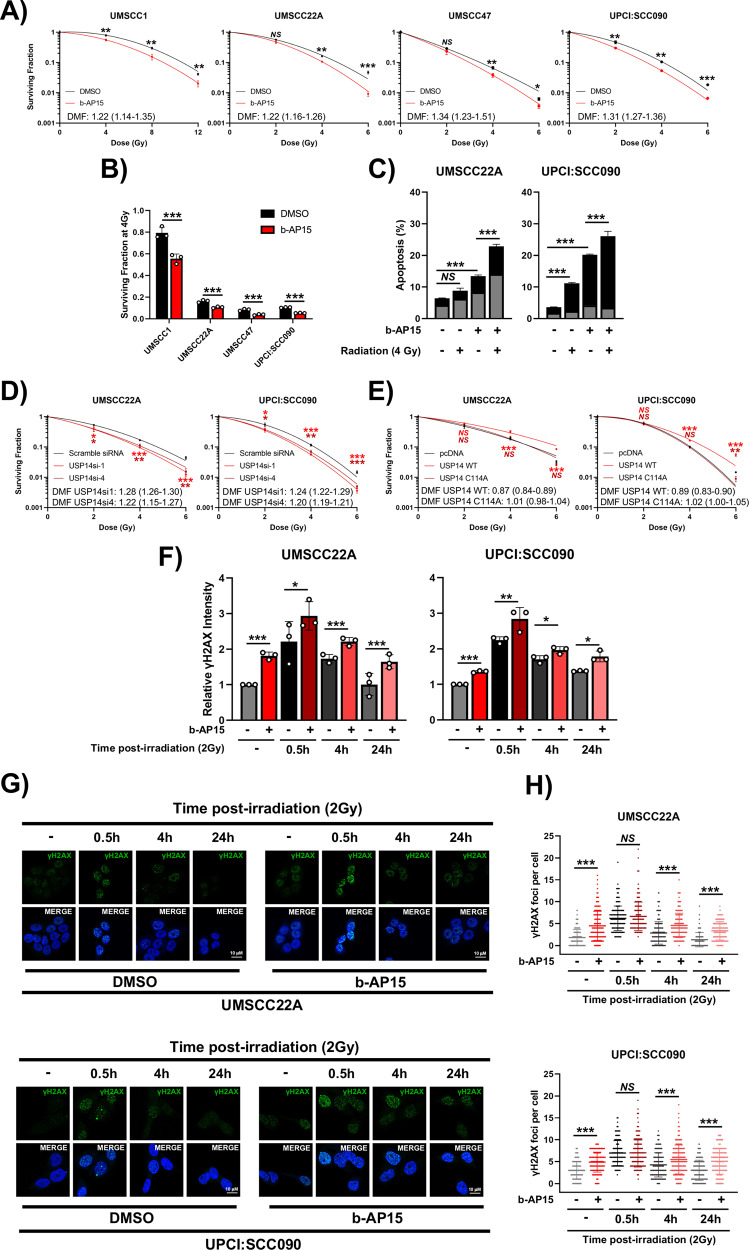
b-AP15 impairs the DNA damage response and sensitizes HNSCC cells to radiation-induced cell death. **A** Clonogenic survival assays of UMSCC1, UMSCC22A, UMSCC47 and UPCI:SCC090 treated with b-AP15 (250 nM) for 2 h before irradiation. Cells were then harvested and replated 24 h after irradiation. **B** Survival fraction at 4 Gy from each cell line in (**A**). **C** Annexin V analysis of UMSCC22A and UPCI:SCC090 cells. Cells were treated with b-AP15 (250 nM) for 2 h before irradiation and assay was performed 24 h after irradiation. **D** Clonogenic survival assays of UMSCC22A and UPCI:SCC090 cells transfected with two specific USP14 siRNAs for 48 h before irradiation. Cells were then harvested and replated 24 h after irradiation. **E** Clonogenic survival assays of UMSCC22A and UPCI:SCC090 cells transfected with USP14 WT and USP14 C11A expression for 24 h before irradiation. Cells were then harvested and replated 24 h after irradiation. **F** UMSCC22A and UPCI:SCC090 cells were treated with b-AP15 (250 nM) for 2 h before irradiation. Cells were then analyzed for γH2AX expression by flow cytometry at the indicated time points. For non-irradiated samples, cells were treated with vehicle or b-AP15 (250 nM) for 24 h before analysis. Data is presented as the relative median fluorescence intensity (MFI) compared to the non-irradiated control samples. **G** Representative immunofluorescence images of γH2AX foci in UMSCC22A and UPCI:SCC090 cells after treatment with b-AP15 (250 nM) and radiation. Cells were treated with b-AP15 (250 nM) for 2 h before irradiation. Cells were then analyzed for γH2AX foci by immunofluorescence analysis at the indicated time points. For non-irradiated samples, cells were treated with vehicle or b-AP15 (250 nM) for 24 h before analysis. **H** Quantification of (**G**). For each condition, foci from at least 200 cells were counted. DMF values were calculated as the vehicle radiation dose for 10% survival divided by radiation dose for 10% survival with the indicated treatment. Bars represent the means ± standard deviation. All experiments are representative of at least three biological replicates. NS not significant; *p  <  0.05; **p  <  0.01; ***p  <  0.001 (Student’s *t* test).

TNFα-dependent radiation-induced cytotoxicity is attenuated by NFκB activity in many cancers, including HNSCC [5, 6]. As our data demonstrated that USP14 inhibition reduces NFκB activity and sensitizes cells to TNFα-induced cell death, we assessed whether HNSCC cells could be sensitized to radiation therapy by USP14 inhibition in vitro. Cells were treated with b-AP15 for 2 h followed by exposure to increasing doses of radiation before clonogenic survival was assessed. Pre-treatment with b-AP15 modestly enhanced the response to radiation treatment in each cell line tested, with dose modification factor (DMF) values ranging from 1.22 to 1.34 (Fig. 7A, B, [48]). Further analysis demonstrated that the combination of b-AP15 and radiation significantly increased apoptosis, 24 h after radiation treatment (Fig. 7C; representative images in Supplementary Fig. 12A). Similar radio-sensitization occurred upon USP14 depletion (Fig. 7D). Furthermore, overexpression of WT USP14, but not the catalytic mutant, increased the resistance to radiation treatment (Fig. 7E).

To further study the observed radiosensitization, we examined the DNA damage response in cells treated with TNF and b-AP15. In control cells, ɣH2AX expression, a marker for dsDNA breaks [49], peaked 30 min post-irradiation and decreased to background levels by 24 h post-irradiation (Fig. 7F; representative images in Supplementary Fig. 12B). In contrast, b-AP15 increased ɣH2AX expression under basal conditions and resulted in increased and prolonged expression up to 24 h post-irradiation. This was confirmed by assessing ɣH2AX foci using immunofluorescence (Fig. 7G, H). In b-AP15 treated cells, radiation-induced ɣH2AX foci were increased in unirradiated cells and were significantly prolonged up to 24 h post-irradiation treatment, suggesting that USP14 may play a role in DNA damage repair in HNSCC cells in vitro.

### b-AP15 delays tumor growth and enhances survival in vivo

**Fig. 8:**
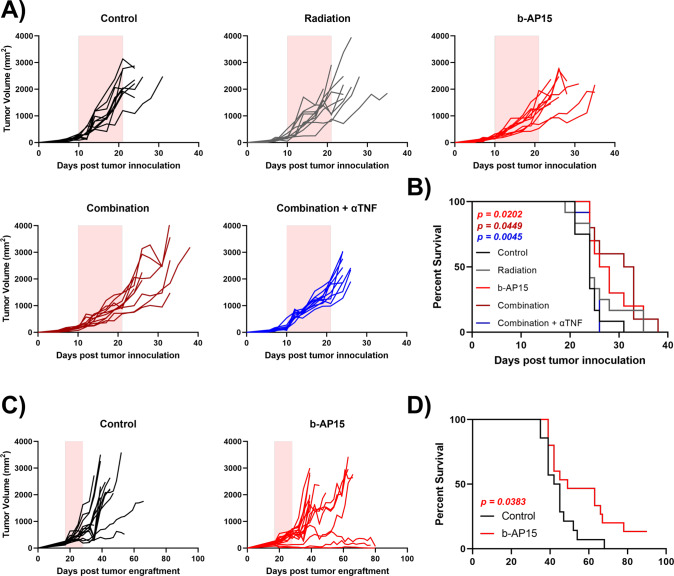
b-AP15 reduced tumor growth and enhanced survival in vivo alone, and in combination with radiation. **A** Individual growth curves of athymic nude mice subcutaneously injected with UMSCC1. Mice were split into 5 groups when the tumor size reached 200 mm^3^; vehicle control, radiation, b-AP15 (5 mg/kg), b-AP15 (5 mg/kg) + radiation or b-AP15 (5 mg/kg) + radiation + anti-mouse TNFα. Each group contain 10–12 mice. The pink shaded area represents the treatment window. **B** Survival curves of the mice in (**A**). **C** Individual growth curves of athymic nude mice subcutaneously injected with UPCI:SCC090. Mice were split into 2 groups when tumor size reached 200 mm^3^; vehicle control, or b-AP15 (5 mg/kg). Each group contain 15 mice. The pink shaded area represents the treatment window. **D** Survival curves of the mice in (**C**). Survival data were plotted using a Kaplan–Meier survival curve, and statistical significance was calculated using the log-rank test.

Next, we investigated the effects of b-AP15 treatment using two separate in vivo models (Supplementary Fig. 13A). For the first study, we chose the HPV- UMSCC1 xenograft mouse model, which is highly resistant to radiation (Fig. 8A). As we observed that b-AP15 sensitized UMSCC1 cells to both TNF- and radiation-induced cell death in vitro, we assessed the ability of b-AP15 to sensitize UMSCC1 to radiation in vivo. b-AP15 alone had a modest but significant inhibitory effect on tumor growth (Fig. 8A). Although the combination of b-AP15 with radiation did not significantly reduce tumor growth further than that observed with b-AP15 alone, median and overall survival was significantly increased in the combination group compared to the control group or radiation alone (both 24 days vs 32 days), and median survival was increased when compared to b-AP15 alone (27 days vs 32 days) (Fig. 8B). Cytokine depletion studies were performed to determine the relative contribution of endogenous mouse TNFα to the observed growth and survival benefit. Depletion of murine TNFα significantly abrogated the anti-tumor effect and survival benefit (24 days vs 33 days) of the combination therapy. These data suggest that b-AP15 reduces tumor growth and significantly enhances survival, both as a monotherapy and potentially in combination with radiation in HPV- xenografts. Furthermore, this suggests that the observed anti-tumor effect and survival benefit in the combination group is at least in part due to host TNF-dependent mechanisms.

Because we observed that b-AP15 also inhibited the viability of HPV + cells, we assessed the activity of b-AP15 monotherapy in a HPV + HNSCC xenograft model, UPCI:SCC090. Treatment resulted in a modest but significant delay in tumor growth (Fig. 8C). Median survival was extended from 43 days to 49 days and overall survival was significantly increased, with one mouse in the treatment group having no detectable tumor at the end of the study (day 90; Fig. 8D). Importantly, b-AP15 treatment had no effect on mouse body weight (Supplementary Fig. 13B) or other apparent toxicities in either mouse study.

## Discussion

Protein ubiquitination is an essential regulatory mechanism that controls several critical cellular processes [18]. Recently, the ubiquitination machinery has emerged as a promising target for cancer treatments [50]. In particular, protein deubiquitinases are attractive targets for small molecule inhibition, because they contain a well-defined active site [51]. The current study provides new data showing that genomic alterations and selective overexpression of the 19S proteasome associated deubiquitinases USP14 and UCHL5 is prevalent in HNSCC. Here, we demonstrate that the high expression of USP14 correlates with worse progression free and overall survival. USP14 inhibition significantly reduced the proliferation and survival of HNSCC cells, sensitizing them to TNFα- induced cell death in vitro.

Previous studies have shown that many deubiquitinases play a role in oncogenesis, including HNSCC and other HPV-associated cancers [52–54]. USP7 promotes radio-resistance through stabilization of TRIP12; increased expression of p16 in HPV + HNSCC results in the degradation of USP7, demonstrating a functional role for a p16/USP7 in promoting radiation sensitivity in HPV + HNSCC [55]. Additionally, BAP1 regulates the response of HNSCC to radiation treatment [52]. Together, these studies demonstrate that therapeutic targeting of protein deubiquitinases and the ubiquitin pathway offers the potential to sensitize HNSCC to current standard-of-care treatments. Treatment options for HNSCC include radio-, chemo- and immune checkpoint therapies, which are critical inducers of the DNA damage response (DDR) and immune responses that can produce cytotoxic TNF-family death ligands [2]. A critical mediator of resistance to these cancer therapies is the canonical NFκB pathway, which promotes the expression of numerous genes involved in proliferation and survival, especially in cancers such as HNSCC [11–13]. Therefore, inhibition of NFκB signalling to sensitize tumors to such treatments warrants further investigation.

Previous efforts aimed at therapeutically inhibiting the NFκB pathway have been complicated by toxicity; therefore, it is vital to identify mechanisms of NFκB activation in order to inhibit its pro-survival effects more selectively. A critical step in the activation of NFκB signalling is the ubiquitination and proteasomal degradation of IκBα [32, 33]. Accordingly, previous studies have demonstrated that proteasome inhibitors such as bortezomib stabilize IκBα, inhibiting RELA nuclear translocation and NFκB-dependent transcription [34, 35, 56]. Proteasome inhibitor (PI) treatment is currently part of the standard of care treatment regimen for Multiple Myeloma (MM), at least in part due to inhibition of NFκB signaling [57]. However, in HNSCC, bortezomib treatment resulted in transient clinical responses, and treatment was limited by neutropenia and other toxicities [34, 56]. Despite the observed inhibition of NFκB activity in correlative studies from these trials, coactivation of other pro-survival signaling pathways, such as STAT3 and MEK/ERK, likely inhibited treatment efficacy [34]. In contrast, b-AP15 treatment reduced NFkB activity without inducing STAT3 or MAPK pathway activation. Whether treatment with USP14 inhibitors results in improved efficacy and less toxicity than that observed with bortezomib in patients requires further clinical study.

Several publications have demonstrated that the anti-cancer efficacy of both PIs and b-AP15 have been attributed, in part, to the inhibition of NFκB activity [34, 49]. However, although the role of USP14 in regulating NFκB activity has been investigated [60–62], a more detailed analysis is lacking. Here, we demonstrate using a wide range of techniques that b-AP15 (and USP14) is a potent inhibitor of TNF-induced NFκB activity, an essential pro-proliferative and pro-survival pathway in HNSCC. Specifically, we demonstrate that inhibition or depletion of USP14 stabilizes IκBα and prevents its degradation. USP14 is a component of the 19S RP that controls ubiquitin recycling and proteasome gate opening [25, 29]. In the case of IκBα, K48-ubiquitination is critical for the activation of NFκB activity by inducing IκBα degradation; this is removed by deubiquitinases including USP15 and USP11, preventing degradation [58, 59]. In contrast, our data suggests that USP14-mediated removal of K48-ubiquitin chains on IκBα is required for its proper degradation, as previously shown [60–62]. This may be due to the function of USP14 in regulating the gate opening of the 20S core of the proteasome, allowing entry of IκBα into the proteasome for efficient degradation, while releasing ubiquitin moieties to maintain the cellular pool.

Previous studies have demonstrated that USP14 is highly expressed in oral cancer and promotes radio-resistance by regulating autophagy [63]. USP14 also regulates the DNA damage response (DDR) by activating autophagy [64, 65]. Other DNA damage associated mechanisms also interact with the NFκB pathway in HNSCC, including WEE1, a G2/M checkpoint kinase that regulates the DDR by preventing damaged DNA entering mitosis [13, 66]. Our data suggest that the reduction of NFκB activity is a critical consequence of USP14 inhibition and that therapeutic inhibition of USP14 in combination with radiation treatment enhances survival in HNSCC xenograft in vivo in a host TNF-dependent manner. As the NFκB pathway also functionally interacts with autophagy signaling [67], further studies are required to investigate whether USP14 alters autophagy in HNSCC.

Similar to other small molecule inhibitors, b-AP15 has several off-target effects. However, a recent study utilizing siRNA screening and RNAseq analysis demonstrated that USP14 depletion resulted in altered gene expression similar to that observed with b-AP15 treatment, further suggesting that USP14 is a critical target of b-AP15 [68]. Our data using other, more specific USP14 inhibitors, as well as USP14 and UCHL5 siRNA, further support this finding. Nonetheless, b-AP15 alters non-USP14 targets, which could result in toxicity. VLX1570, a derivative of b-AP15, was designed to improve its specificity and ‘drug-like’ properties of b-AP15. Despite promising pre-clinical data, a recent phase I trial was terminated owing to significant lung toxicities [69].

In summary, our data demonstrate that USP14 plays a key role in the activation of NFκB activity and the response to radiation therapy in HNSCC cells. This effect seems to be independent of HPV status and may be clinically relevant, as USP14 expression correlates with poor progression free survival in HPV- HNSCC. Our in vivo data demonstrate that targeting USP14 may have therapeutic benefits in combination with TNFα-inducing therapies such as radiation in HNSCC. Further pre-clinical and clinical development of small molecules targeting USP14 is warranted.

## Materials and methods

### HNSCC cell lines

A panel of HNSCC cell lines from the University of Michigan squamous cell carcinoma (UMSCC) series was obtained from Dr. T.E. Carey. These UMSCC and UPCI cell lines were authenticated by genotyping with 9 markers and by whole exome sequencing [40]. Cells were cultured in minimal essential medium (MEM) supplemented with 10% fetal calf serum, penicillin and streptomycin (100 µg/mL), and MEM non-essential amino acids (NEAA), and used for fewer than 15 passages. Human primary oral keratinocytes (HOK) from oral gingival mucosa were purchased from Science Cell Research laboratories and used as a control cell line. The cells were cultured in serum-free oral keratinocyte medium with supplements (Science Cell) for fewer than four passages.

### In vivo tumor studies

All in vivo experiments N/NIH Animal Care and Use Committee under protocol #1322, in compliance with the Guide for the Care and Use of Laboratory Animal Resource National Research Council. 4 to 6-week-old female athymic nu/nu mice were obtained from Taconic Biosciences and housed in a pathogen-free NIH animal facility. Cells were mixed with Matrigel (R&D Systems) in a 70% cells :30% Matrigel solution. Each mouse was subcutaneously injected in the right thigh (5 × 10^6^ cells). Tumor volumes were measured 2–3 times weekly, and mice were randomized into the respective groups when tumor volumes reached ~200 mm^3^. For UMSCC1 tumor xenografts, mice were divided into five groups; vehicle control, b-AP15 (5 mg/kg), radiation (20 Gy total; 10 × 2 Gy) or combination (b-AP15 (5 mg/kg) plus radiation (10 × 2 Gy)). An additional group of mice was treated with b-AP15 (5 mg/kg) plus radiation (10 × 2 Gy) with the addition of anti-mouse TNFα antibody (200 μg per mouse per dose; XT3.11, BioCell) 7 times, starting 3 days before treatments began, and subsequently 3 times per week for the 2 weeks (Supplementary Fig. 6). For UPCI:SCC090 tumor xenografts, mice were randomised into 2 groups: vehicle control (Cremophor EL and polyethylene glycol 400 (1:1) 0.9% saline) or b-AP15 (5 mg/kg). Mouse body weight, body condition, and tumor size was recorded two or three times a week for the indicated days. The tumor volume was calculated with the formula V = ½ L*W2, and tumor growth was reported as the mean tumor volume ± standard error. Kaplan-Meier survival analysis was performed using the GraphPad PRISM software (v6.05). Survival statistics were performed using the log-rank test.

### Analyses of publicly available datasets

Analysis of gene and protein expression in HNSCC cases was performed using publicly available data from TCGA (https://portal.gdc.cancer.gov/repository) [15] and the Gene Expression Omnibus (GEO; GSE6791 and GSE25099) [70, 71].

### Statistical analysis

Cell viability was analyzed for synergy using CompuSyn [47]. Patient overall survival data were downloaded from the Firehose at the Broad Institute (https://confluence.broadinstitute.org/display/GDAC/Home/). Patient progression-free survival data were downloaded from the Supplement Information of a pan-cancer clinical study [38]. The clinical survival endpoints for patients were progression-free survival (PFS), and overall survival (OS). PFS is the time from the date of diagnosis until tumor progression or death, whichever occurs first. OS is the period from the date of diagnosis until the date of death from any cause. The PFS and OS curves were obtained using Kaplan-Meier method and were compared using the log-rank test. The Cox proportional hazards model was used to estimate Hazard Ratios (HRs) with 95% Confidence Intervals (CIs).

For differential gene expression analysis between normal and tumor tissue, and HPV- and HPV + tumor tissue, Wilcoxon sum rank test was used to assess statistical significance. All graphs were prepared using the GraphPad Prism (GraphPad, USA) or R software version 4.0.2. Combination index was calculated using CompuSyn. Error bars represent mean ± the standard error. Statistical significance was determined as follows: NS = not significant, *p < 0.05, **p < 0.01, ***p < 0.001.

## Supplementary information

Supplementary Figures

Supplementary Table 1 and 2

Supplementary Table 3

Supplemental Materials and Methods

Uncropped Blots

Author Contribution List

Reproducibility Checklist

## Data Availability

All data is included within the main text and supplementary files.
