# Supplementary material for: Inhibition of USP14 promotes TNFα-induced cell death in head and neck squamous cell carcinoma (HNSCC)
Source: Cell Death Differ. 2023 Apr 13;30(5):1382–96. doi: 10.1038/s41418-023-01144-x (PMC10154301; doi:10.1038/s41418-023-01144-x)
Supplement: Supplementary file 1 — Supplementary Figures [file 41418_2023_1144_MOESM1_ESM.pdf]

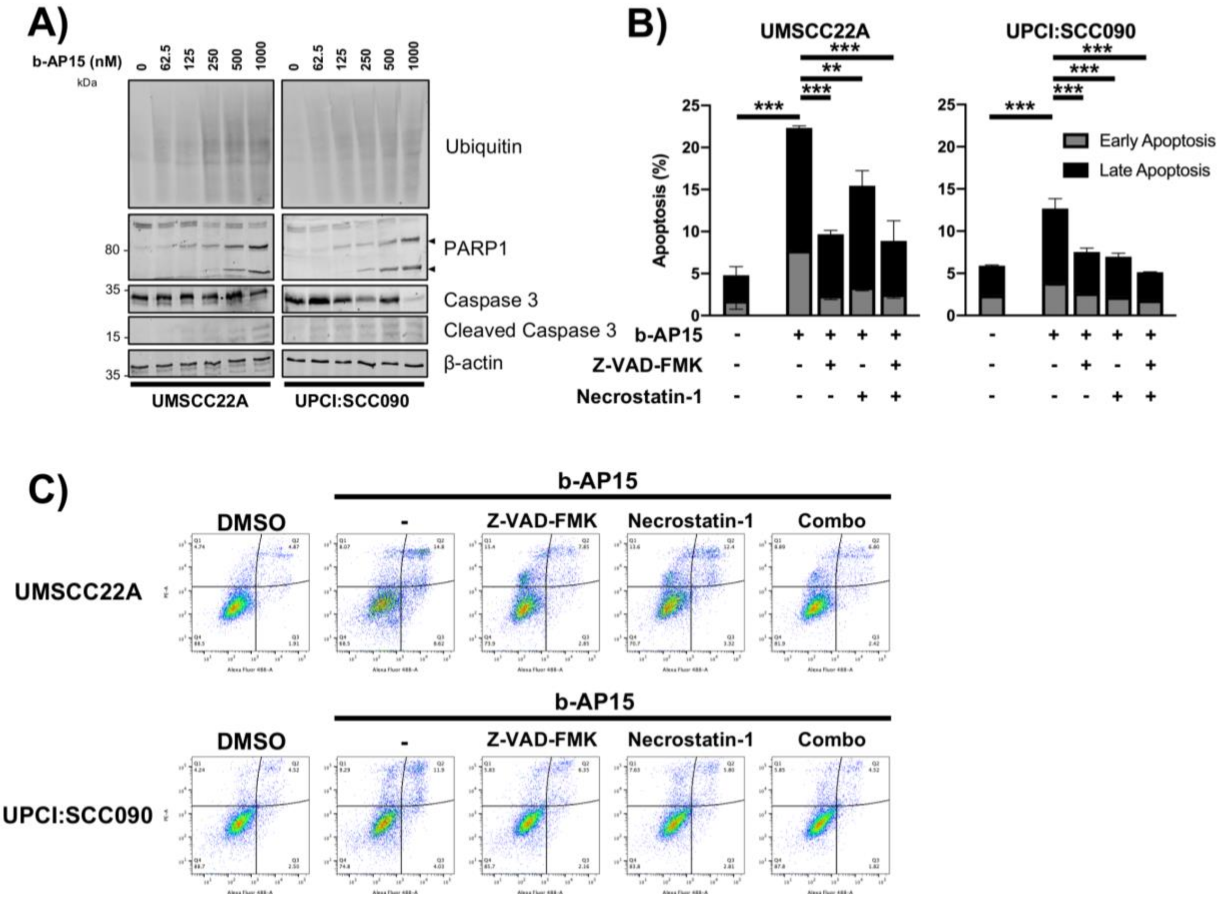

Supplementary Figure 1. b-AP15 induces accumulation of polyubiquitination and caspase- and RIPK1-dependent apoptosis. A) Representative western blot of UMSCC22A and UPCI:SCC090 cells after treatment with increasing doses of b-AP15 for 24h. Lysates were analyzed for Ubiquitin, PARP1 and Caspase 3 cleavage. β-actin was used as the loading control. **B)** Annexin V analysis of UMSCC22A and UPCI:SCC090 cells after treatment with b-AP15 for 24h. Prior to treatment, cells were treated with the pan-caspase inhibitor Z-VAD-FMK and/or the RIPK1 inhibitor necrostatin-1. **C)** Representative images for data in **B)**. Bars represent the means ± standard deviation. All experiments are representative of at least three biological replicates. *NS* – not significant; *p < 0.05; **p < 0.01; ***p < 0.001 (Student's t-test).

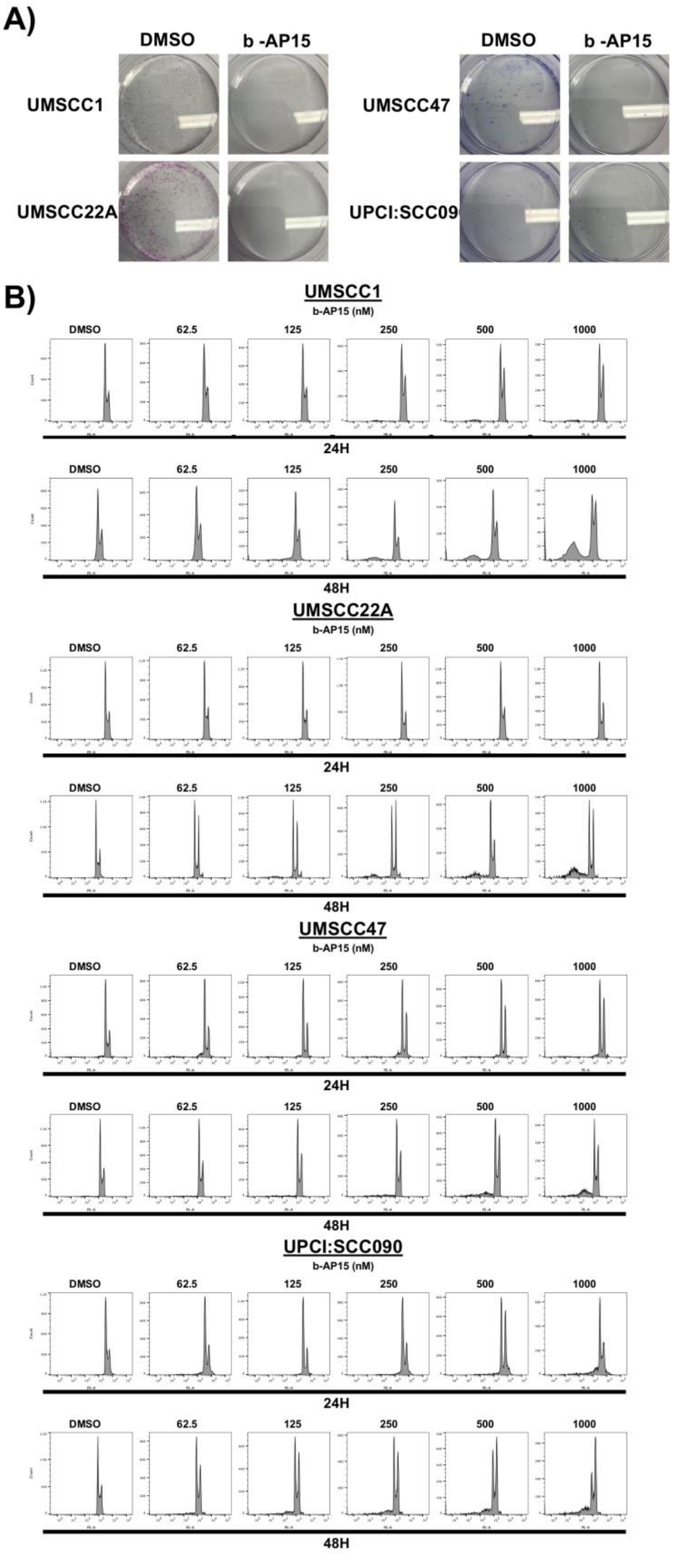
A)
DMSO
b -AP15
UMSCC1
UMSCC22A
UMSCC47
UPCI:SCC09
B)
UMSCC1
b-AP15 (nM)
DMSO
62.5
125
250
500
1000
24H
48H
UMSCC22A
24H
48H
UMSCC47
24H
48H
UPCI:SCC090
24H
48H

Supplementary Figure 2. Representative images from Figure 2. A) Representative images of colony formation assays in Figure 2D. **B)** Representative images of cell cycle analysis in Figure 2E.

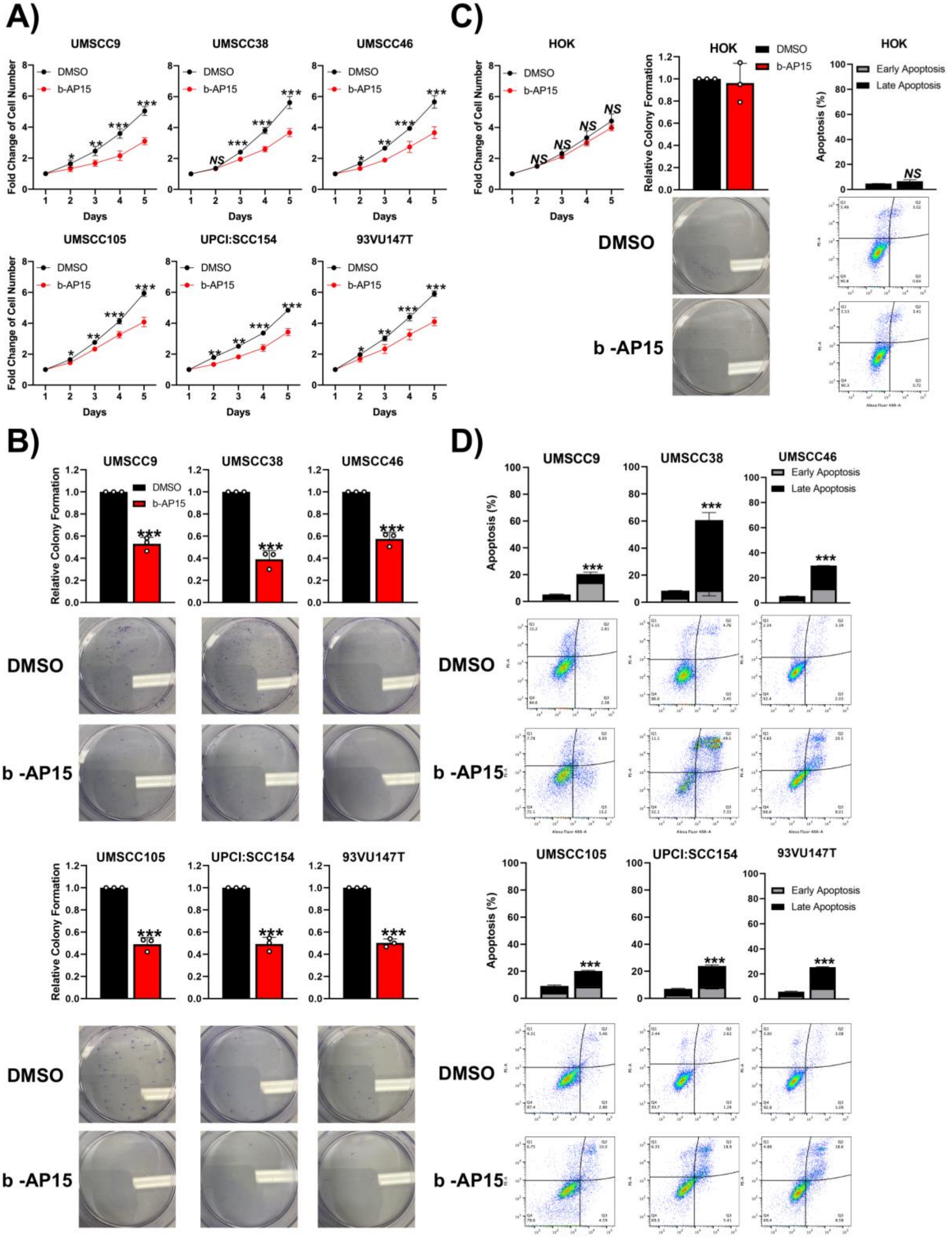
A)
UMSCC9
UMSCC38
UMSCC46
UMSCC105
UPCI:SCC154
93VU147T
Fold Change of Cell Number
Days
DMSO
b-AP15
C)
HOK
Relative Colony Formation
Apoptosis (%)
Early Apoptosis
Late Apoptosis
NS
B)
D)
DMSO
b -AP15
Alexa Fluor 488-A

Supplementary Figure 3. The effects of b-AP15 on a wider panel of HNSCC cells. A-C) Cell growth analysis **(A)** and colony formation assay **(B)** of UMSCC9, UMSCC38, UMSCC46, UMSCC105, UPCI:SCC154 and 93VU147T 24h after b-AP15 (250 nM) treatment. Representative images are included below each graph. **C)** Cell growth, colony formation and annexin assay of HOK cells after treatment with b-AP15 (2500 nM) for 24h. Representative images are included below each graph. **D)** Annexin V analysis of UMSCC9, UMSCC38, UMSCC46, UMSCC105, UPCI:SCC154 and 93VU147T 24h after b-AP15 (250 nM) treatment. Representative images are included below each graph. Bars represent the means ± standard deviation. All experiments are representative of at least three biological replicates. *NS* – not significant; *$p < 0.05$; **$p < 0.01$; ***$p < 0.001$ (Student's t-test).

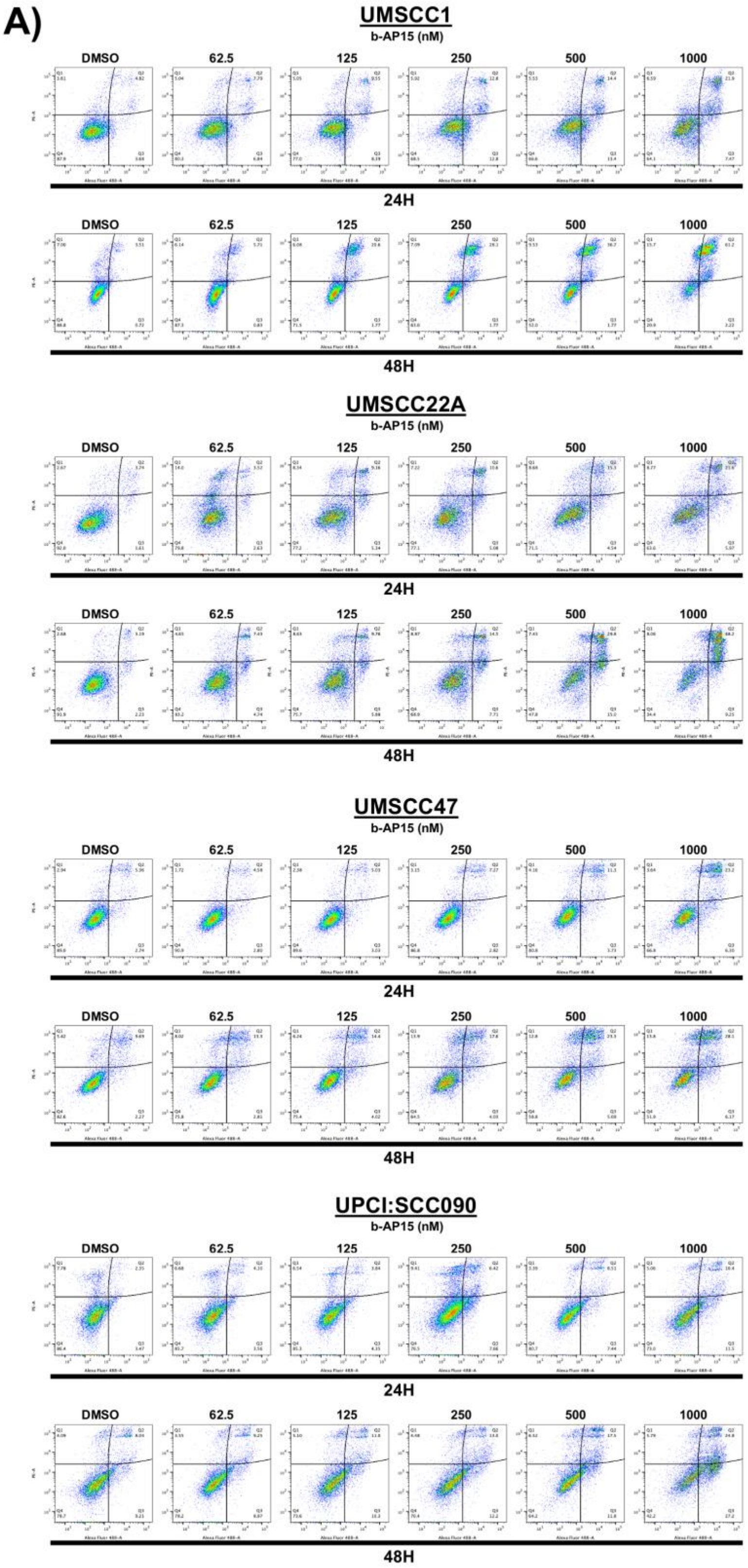

Supplementary Figure 4. Representative images from Figure 2. A) Representative images of Annexin V assay in Figure 2F.

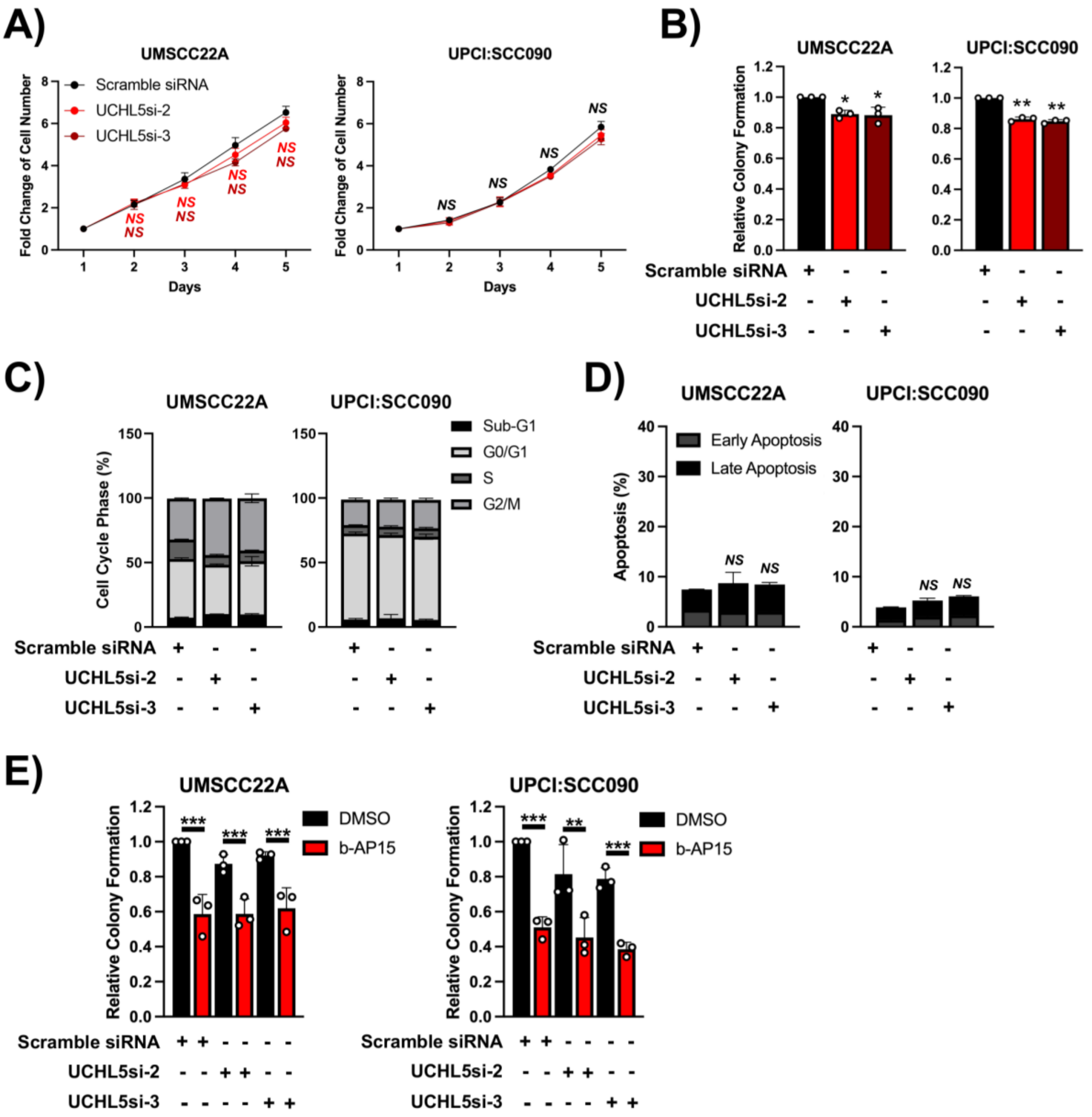

Supplementary Figure 5. UCHL5 depletion has minimal impact in HNSCC cells. A-C) Cell growth analysis **(A),** colony formation assay **(B),** cell cycle analysis **(C)** and Annexin V analysis **(D)** of UMSCC22A and UPCI:SCC090 cells after transfection with two specific UCHL5 siRNAs for 72 h. **E)** Colony formation assay of UMSCC22A and UPCI:SCC090 cells after transfection of two

specific UCHL5 siRNAs for 72 h. After 48h, cells were additionally treated with b-AP15 (250 nM) or vehicle control. Bars represent the means ± standard deviation. All experiments are representative of at least three biological replicates. *NS* – not significant; *p < 0.05; **p < 0.01; ***p < 0.001 (Student's t-test).

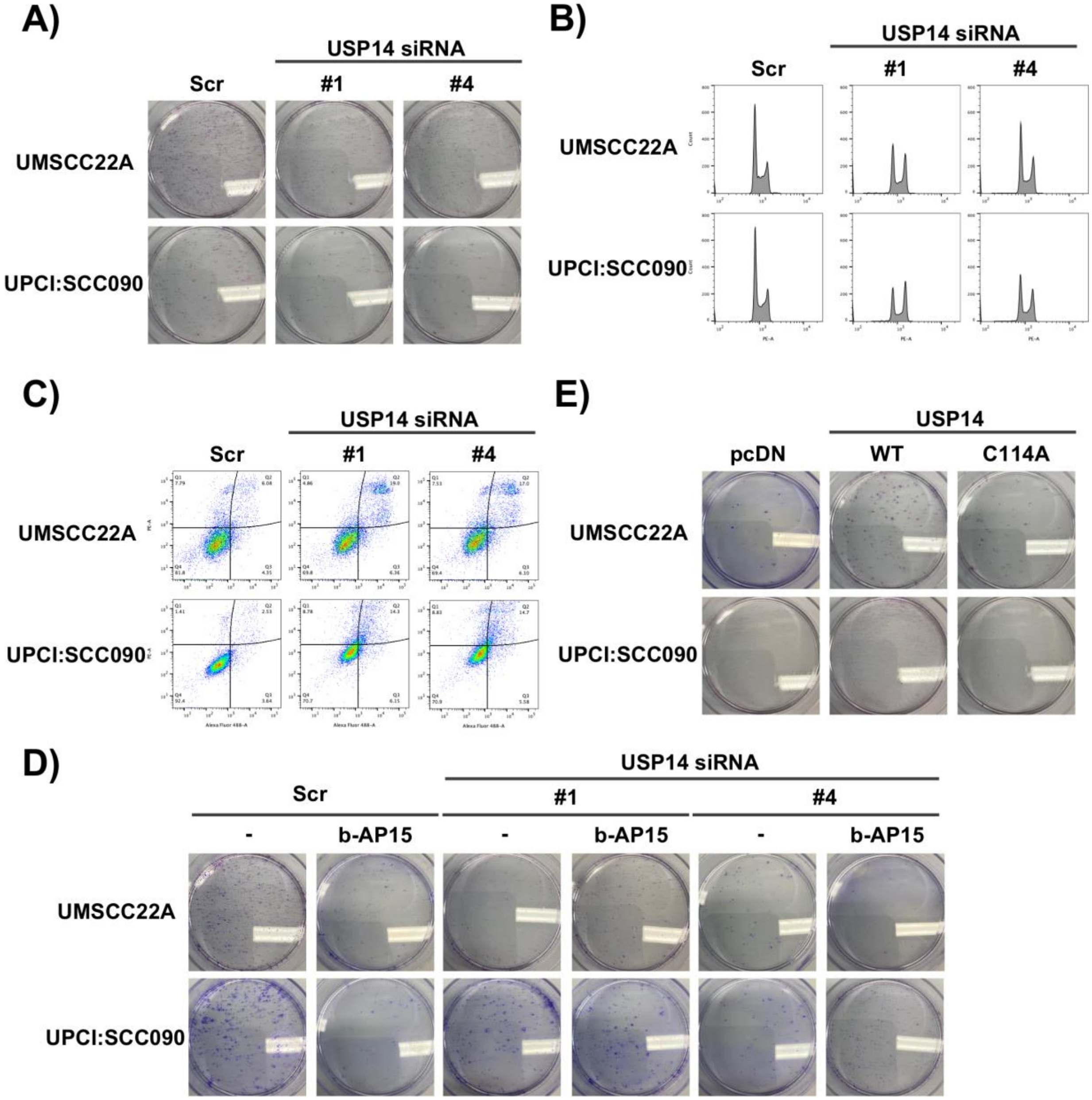

Supplementary Figure 6. Representative images from Figure 3. A) Representative images of colony formation assays in Figure 3C. **B)** Representative images of cell cycle analysis in Figure 3D. **C)** Representative images of Annexin V assay in Figure 3E. **D)** Representative images of colony formation assays in Figure 3F. **E)** Representative images of colony formation assays in Figure 3I.

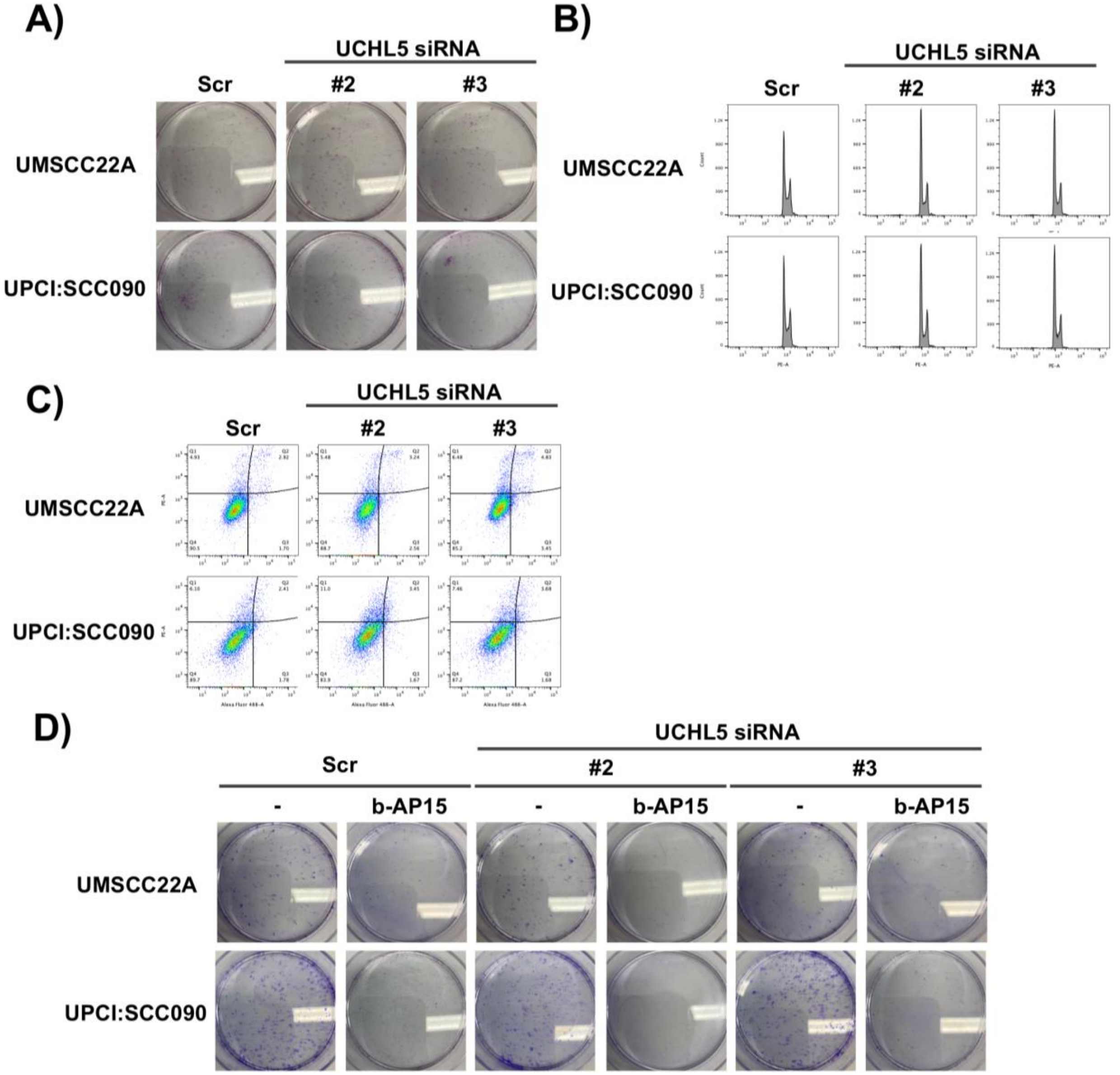

Supplementary Figure 7. Representative images from Supplementary Figure 5. A) Representative images of colony formation assays in Supplementary Figure 5B. **B)** Representative images of cell cycle analysis in Supplementary Figure 5C. **C)** Representative images of Annexin V assay in Supplementary Figure 5D. **D)** Representative images of colony formation assays in Supplementary Figure 5E.

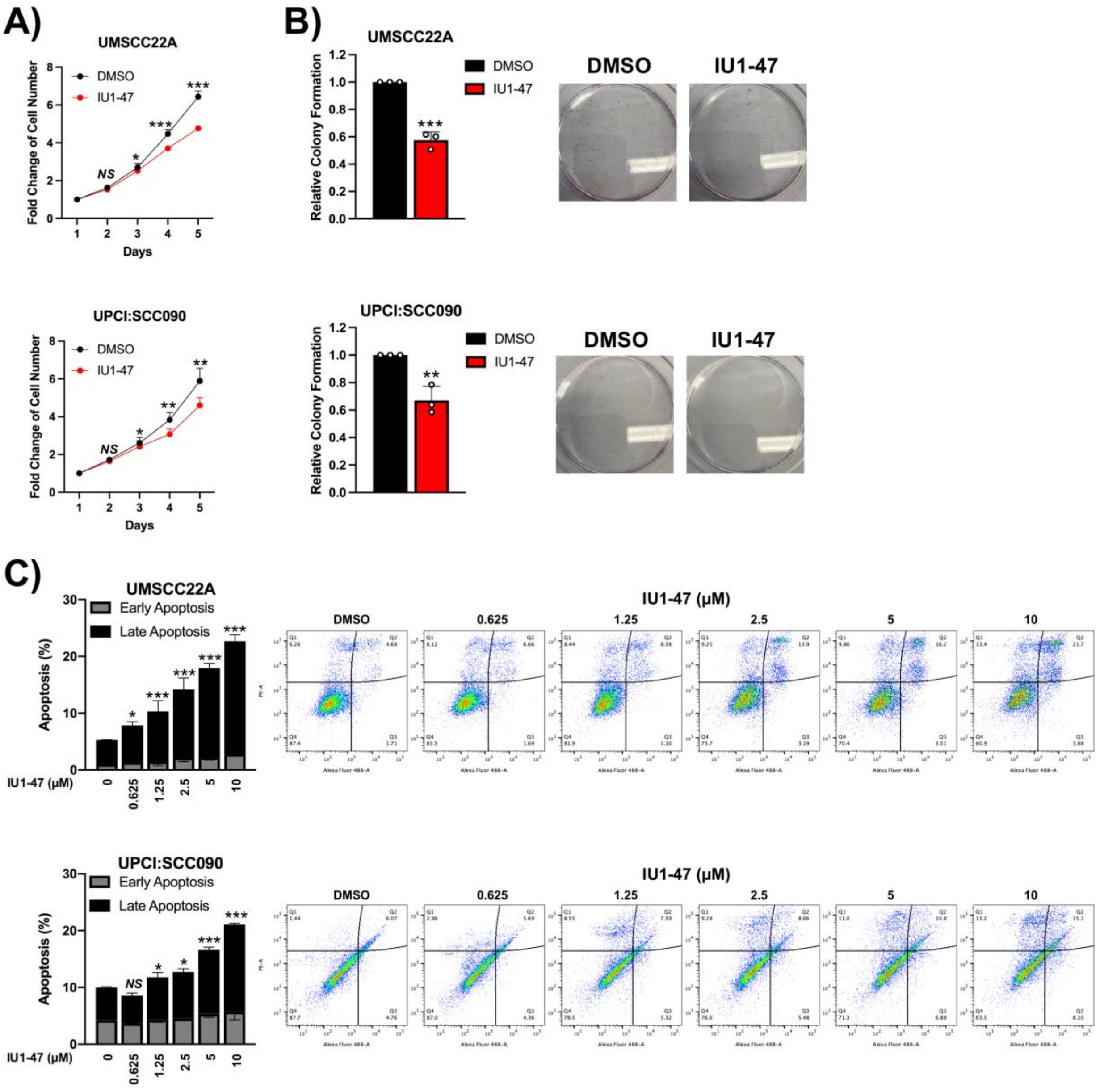

Supplementary Figure 8. Specific inhibition of USP14 reduces proliferation and induces apoptosis in HNSCC cells. A-C) Cell growth analysis **(A)**, colony formation assay **(B)** and Annexin V analysis **(C)** of UMSCC22A and UPCI:SCC090 cells after treatment with IU1-47 (2.5 μM) for 24 h. Representative images are included next to each graph. Bars represent the means ± standard deviation. All experiments are representative of at least three biological replicates. *NS* – not significant; *p < 0.05; **p < 0.01; ***p < 0.001 (Student's t-test).

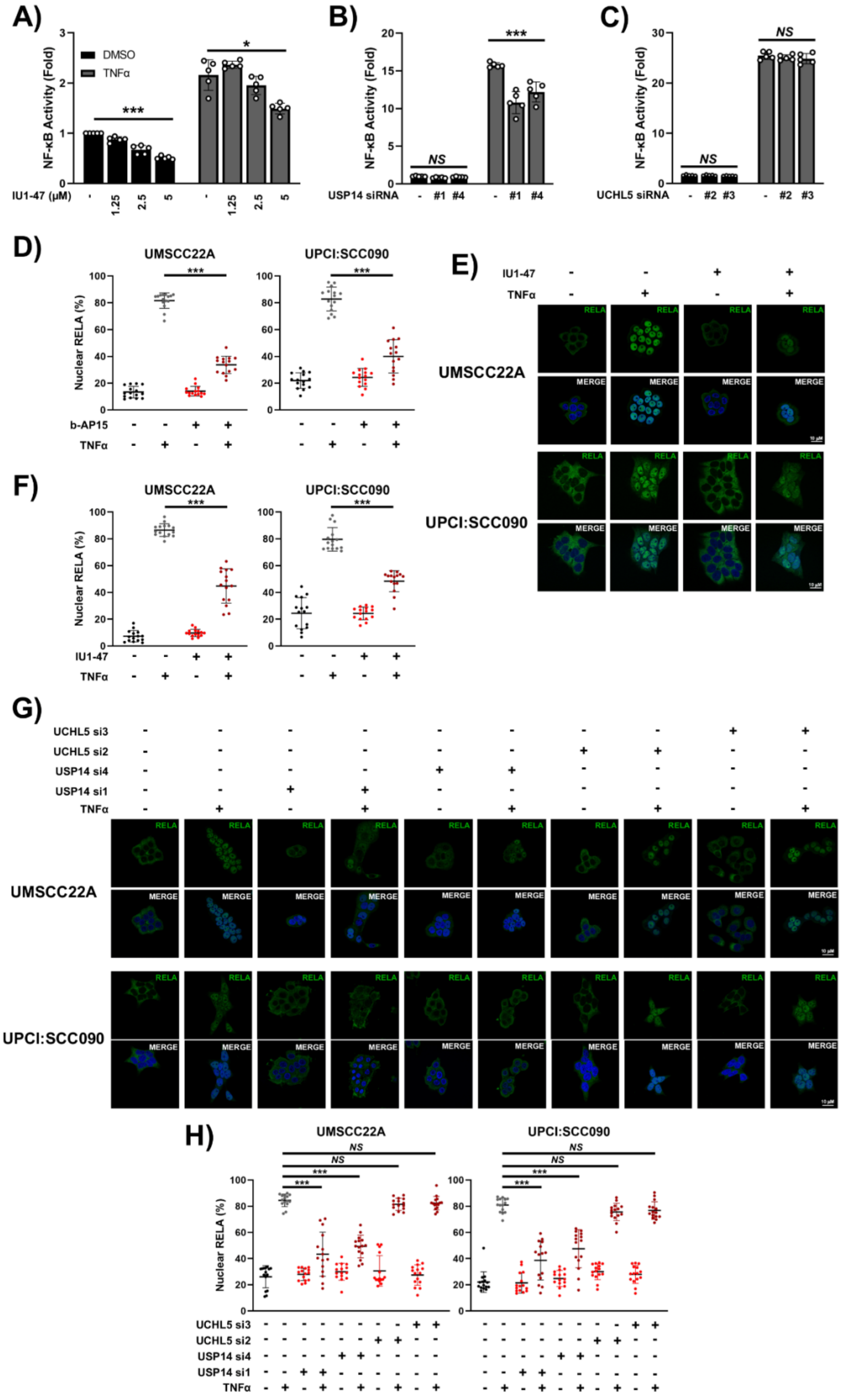

A)
B)
C)
D)
E)
F)
G)
H)
NF-κB Activity (Fold)
DMSO
TNFα
IU1-47 (μM)
USP14 siRNA
UCHL5 siRNA
NS
UMSCC22A
UPCI:SCC090
Nuclear RELA (%)
b-AP15
IU1-47
RELA
MERGE
UCHL5 si3
UCHL5 si2
USP14 si4
USP14 si1

Supplementary Figure 9. Inhibition of USP14 reduces NFκB activity and RELA nuclear translocation in HNSCC cells. A) NFκB reporter activity after treatment with increasing doses of IU1-47. Cells were treated with IU1-47 or vehicle control for 24h, with TNFα (20 ng/mL) added for the final 16h. **B-C)** NFκB reporter activity after transfection with two specific USP14 **(B)** or UCHL5 **(C)** siRNAs for 72h, with TNFα (20 ng/mL) added for the final 16h. **D)** Quantification of percentage nuclear RELA from Figure 4E. Data represents the percentage nuclear localisation of RELA from 15 cells and was analyzed using ImageJ as previously (17). **E)** Representative immunofluorescence images of RELA localisation. UMSCC22A and UPCI:SCC090 cells were treated with IU1-47 (2.5 μM) or vehicle control for 6h, with TNFα (20 ng/mL) added for 30 min. DAPI was used as a nuclear counterstain. **F)** Quantification of percentage nuclear RELA from **E)**. Data represents the percentage nuclear localisation of RELA from 15 cells and was analyzed using ImageJ as previously (17). **G)** Representative immunofluorescence images of RELA localisation. UMSCC22A and UPCI:SCC090 cells transfection with two specific USP14 or UCHL5 siRNAs for 72 h, with TNFα (20 ng/mL) added for the final 30 min. DAPI was used as a nuclear counterstain. **H)** Quantification of percentage nuclear RELA from **G)**. Data represents the percentage nuclear localisation of RELA from 15 cells and was analyzed using ImageJ as previously (17). Bars represent the means ± standard deviation. All experiments are representative of at least three biological replicates. *NS* – not significant; *p < 0.05; **p < 0.01; ***p < 0.001 (Student's t-test).

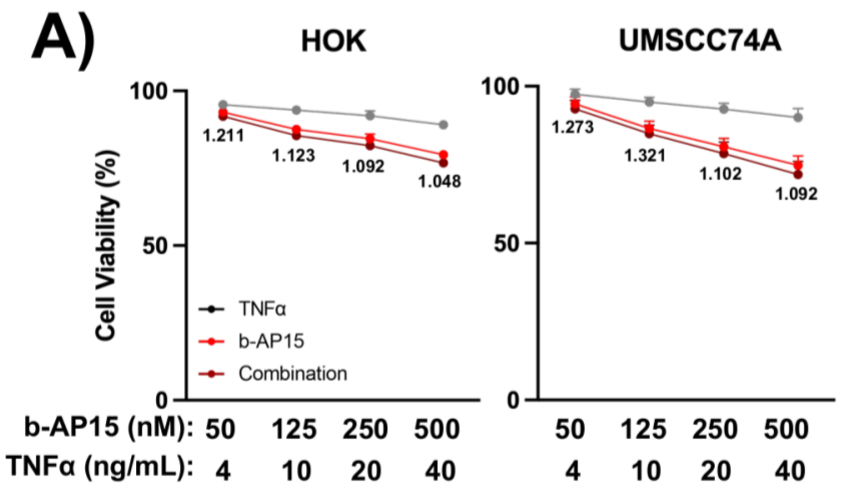

Supplementary Figure 10. b-AP15 does not sensitizes WT TP53 cells to TNFα-induced cell death. A) XTT cell viability analysis of HOK and UMSCC74A cells with varying doses of b-AP15 and TNFα for 48h. Values below the combination are Combination Indices (CI) as described in the text. Bars represent the means ± standard deviation. All experiments are representative of at least three biological replicates.

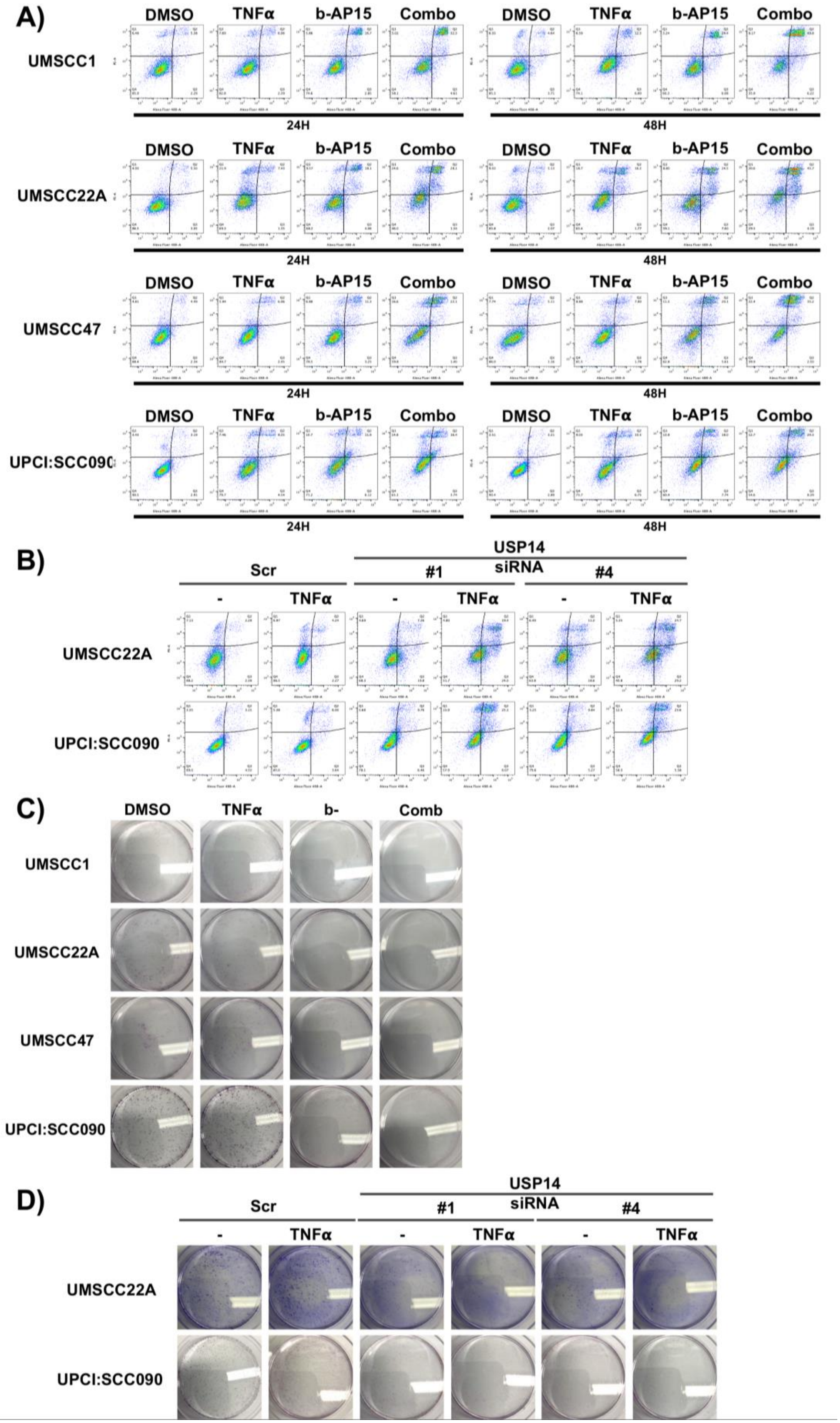
A)
DMSO
TNFα
b-AP15
Combo
UMSCC1
UMSCC22A
UMSCC47
UPCI:SCC090
24H
48H
B)
USP14
siRNA
Scr
#1
#4
-
TNFα
UMSCC22A
UPCI:SCC090
C)
DMSO
TNFα
b-
Comb
UMSCC1
UMSCC22A
UMSCC47
UPCI:SCC090
D)
USP14
siRNA
Scr
#1
#4
-
TNFα
UMSCC22A
UPCI:SCC090

Supplementary Figure 11. Representative images from Figure 6. A) Representative images of Annexin V assay in Figure 6B. **B)** Representative images of Annexin V assay in Figure 6D. **C)** Representative images of colony formation assays in Figure 6E. **D)** Representative images of colony formation assays in Figure 6F.

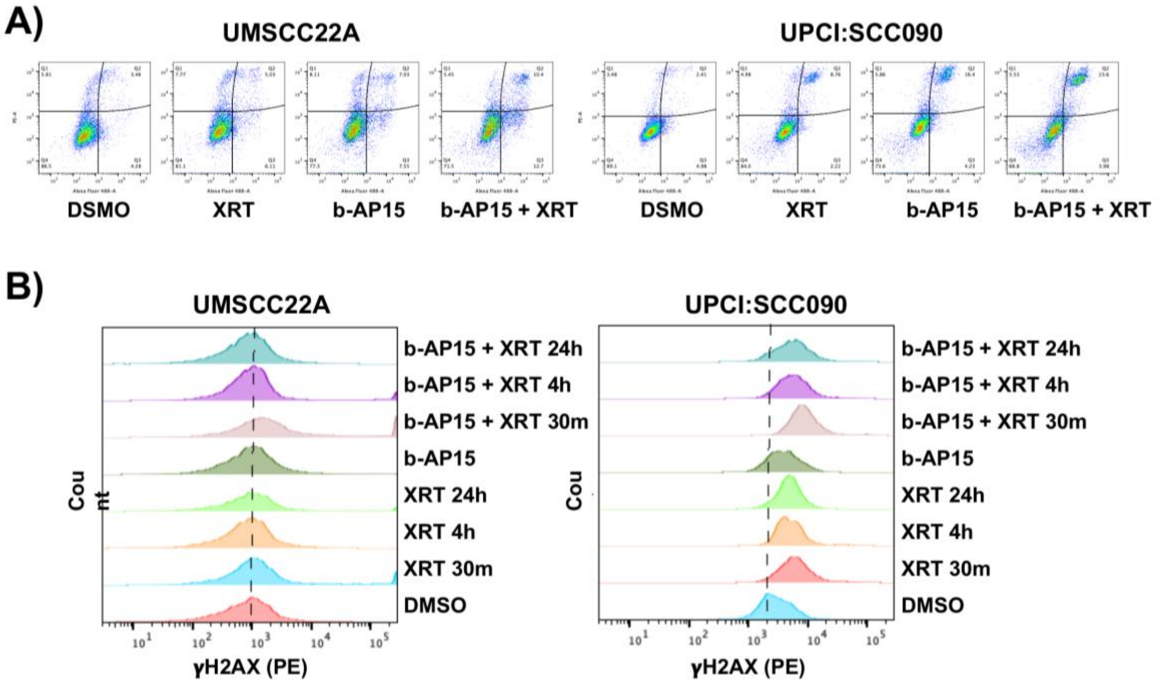

Supplementary Figure 12. Representative images from Figure 7. A) Representative images of Annexin V assay in Figure 7C. **B)** Representative images of γH2AX intensity in Figure 7F.

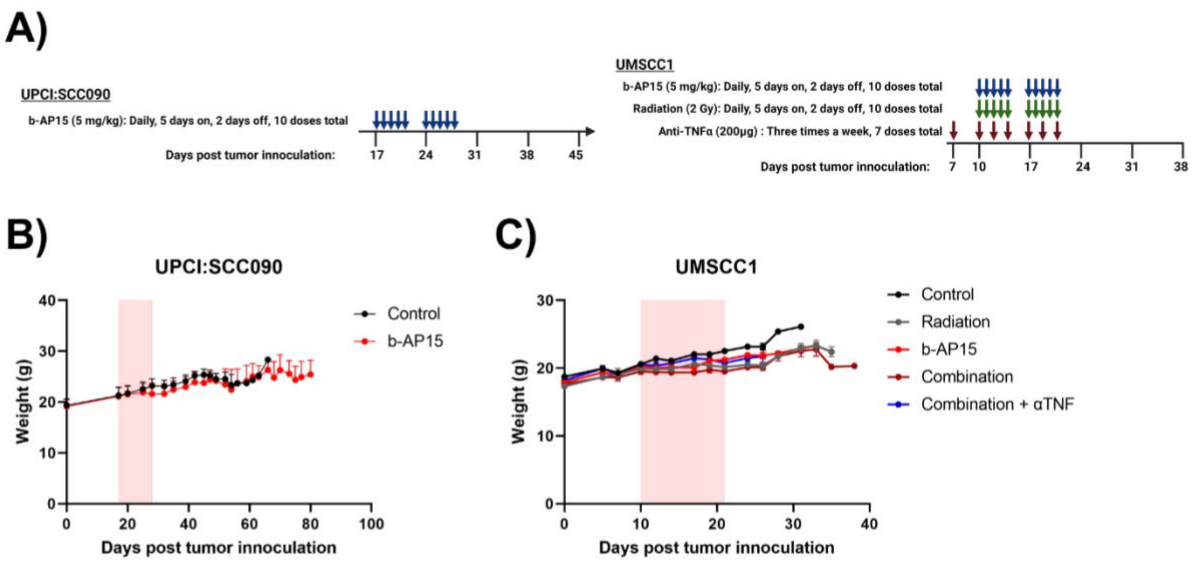

Supplementary Figure 13. b-AP15 has minimal impact on mouse weight *in vivo*. A) Schematic of *in vivo* experiments in Figure 8. **B)** Mouse weights during the experiment in (Figure 8A-B)**. C)** Mouse weights during the experiment in (Figure 8C-D)**.**
