# Supplementary material for: Inhibition of USP14 promotes TNFα-induced cell death in head and neck squamous cell carcinoma (HNSCC)
Source: Cell Death Differ. 2023 Apr 13;30(5):1382–96. doi: 10.1038/s41418-023-01144-x (PMC10154301; doi:10.1038/s41418-023-01144-x)
Supplement: Supplementary file 4 — Supplemental Materials and Methods [file 41418_2023_1144_MOESM4_ESM.docx]

**Supplemental Materials and Methods
Plasmids, siRNA and reagents**
Plasmids for FLAG-USP14, HA-RELA and HA-IkBα were purchased from Origene. The USP14 catalytic mutant USP14 C114A was made by Origene. The HA-Ubiquitin plasmid and the HA-Ubiquitin mutants were purchased from the University of Dundee MRC PPU Reagents depository. siRNA targetting USP14 were purchased from Qiagen (FlexiTube GeneSolution GS9097 for USP14; SI00072982 and SI00072961). siRNA targetting UCHL5 were purchased from Qiagen (FlexiTube GeneSolution GS51377 for UCHL5; SI05707912 and SI05707905). siRNA was used at 50nM. The small molecule inhibitors b-AP15, IU1-47, Bortezomib and Cycloheximide were purchased from MedChemExpress. All drugs were used at the indicated concentrations and incubation periods listed in the figure legend. Human recombinant TNFα (210-TA) was purchased from R&D Systems and used at 20 ng/μL.

**Radiation experiments**In vitro and in vivo experiments were irradiated with separate XRAD320 X-ray irradiators (Precision X-ray, Inc., North Branford, CT) housed in the Radiation Biology Branch and Animal Care Facility, respectively, of the National Cancer Institute. Unanesthetized mice were held in custom plexiglass jigs shielded with lead leaving only the right tumor-bearing hind leg exposed to ionizing radiation. For all experiments, ionizing radiation was delivered at a dose rate of ~2.42 Gy/min with 300kV X-rays at a distance of ~50cm from the radiation source.

**Tissue Microarray Analysis**
A human oral squamous cell cancer TMA containing 50 cases of oral squamous cell cancer and 10 cases of normal oral tissue (OR601c) and a head and neck TMA containing 70 cases of head and neck cancer and 10 cases of normal oral tissue (HN802c) were purchased from GeneTex, Inc.. Slides were deparaffinized in xylene, rehydrated in a graded series of ethanol solutions and subjected to antigen retrieval in citric acid. Slides were blocked in normal serum and incubated in primary antibody (USP14 (D8Q6S; 11931, CST) overnight at 4 °C. Slides were then processed using the VECTASTAIN® Universal Quick HRP Kit (PK-7800; Vector Laboratories) as per the manufacturer’s instructions. Immunostaining was visualized using 3,3’-diaminobenzidine (Vector® DAB (SK-4100; Vector Laboratories)). USP14 immunostaining quantification was automated using ImageJ with the IHC Profiler plug-in (Ref). Histology scores (H-score) were calculated based on the percentage of positively stained tumour cells and the staining intensity grade. The staining intensities were classified into the following four categories: 0, no staining; 1, low positive staining; 2, positive staining; 3, strong positive staining. H score was calculated by the following formula: (3 × percentage of strong positive tissue) + (2 × percentage of positive tissue) + (percentage of low positive tissue), giving a range of 0–300.
 **siRNA depletion**
UMSCC22A or UPCI:SCC090 cells were plated as required and transfected with siRNA targetting USP14, UCHL5 or negative control siRNA (Dharmacon, Cat#D-001810-0X) at a final concentration of 50nM using Lipofectamine 2000 following the manufacturer’s protocol (Life Technologies, Cat#13778150). At 72h post transfection, cells were harvested for analysis as required.

**NFκB reporter assays**A stable reporter line, UMSCC1^κB^ cells previous developed in our lab (13), were treated as indicated in the figure legends. Relative NFκΒ activity (blue/green fluorescent ratio) was then observed via plate reader.

 **Western blot**
 Cells were treated as indicated in the figure legends. cells were washed with cold 1X PBS, trypsinised and resuspended in lysis buffer (10 mM Tris, pH7.5, 0.5% Triton X-100, 150 mM NaCl, 0.5 mM EDTA) containing protease and phosphatase inhibitor cocktail (HALT protease and phosphatase inhibitor cocktail, Thermo Scientific, Waltham, MA). For cellular fraction, after trypsinization cell were processed using the Nuclear Extraction Kit (Abcam, ab113474). Protein concentrations were determined using the Pierce BCA Protein Assay Kit (Thermo Scientific). Lysates for each lane were then loaded on SDS-PAGE gels and transferred to nitrocellulose membranes using the Invitrogen iBlot 2 system, according to the manufacturer’s standard protocol. After blocking in Odyssey blocking buffer (Li-COR Biosciences, Lincoln, Nebraska USA), membranes were incubated with primary antibodies overnight at 4°C. After washing, membranes were incubated with species-specific IRdye-conjugated secondary antibodies for 1 hour at room temperature. Signal was visualized using LI-COR ODYSSEY Infrared Imaging System (Li-COR Biosciences). The following antibodies were used at 1:1000 dilution unless otherwise stated: USP14 (1:2000, D8Q6S; 11931, Cell Signalling Technology (CST)), IkBα (L35A5; 4814, CST), pIKKα/β (S176/S177 – 1:500; 2078, CST), IKKα (11930, CST), IKKβ (8943, CST), pRELA (S536; 3033, CST), RELA (8242, CST), RELB (1:500; 4922, CST), NFκB1 p105/p50 (13586, CST), TP53 (sc-126, Santa Cruz Biotechnology (SCBT)), phospho-ERK1/2 (Thr202/Tyr204) (4370, CST), ERK1/2 (4695, CST), phospho-STAT3 (Y705) (9131), STAT3 (9139, CST), PARP (9532, CST), Cleaved Caspase 3 (N175 – 1:500; 9664, CST), Caspase 3 (14220, CST), γH2AX (9718, CST), FLAG (14793, CST and β-actin (1:5000, sc-47778, SCBT).

**Co-immunoprecipitation assay (Co-IP)**

Co-IP experiments were performed using Pierce™ Crosslink Magnetic IP/Co-IP Kit (Thermo Fisher Scientific, USA) following the manufacturer's protocol, except whole-cell lysates were collected using lysis buffer (1% Triton X-100, 0.5% deoxycholate, 1% NP-40, 50 mM Tris, 100 mM NaCl, 2 mM MgCl2, 10% glycerol). For each IP, 50 µg protein was incubated with 5 µL antibody: USP14 (D8Q6S; 11931, Cell Signalling Technology (CST)), IkBα (L35A5; 4814, CST), IKKα (11930, CST), IKKβ (8943, CST), IKKγ (2685, CST), RELA (3039, CST), RELB (4922, CST) and NFκB1 p105/p50 (13586, CST). For input lysates, 20 µg protein was loaded and the input and immunoprecipitated proteins were then analyzed by SDS-PAGE and Western blot. Western blot was performed as described in the supplementary methods. Non-immune rabbit IgG or mouse IgG was included as a negative control.
 **Ubiquitination assays**

For exogenous ubiquitination assays, FLAG-USP14 and HA-ubiquitin plasmids were transfected into UMSCC22A cells using Lipofectamine 2000 (Invitrogen). After 40 h, cells were treated with 100 nM bortezomib (MedChemExpress) for 8 h. For endogenous ubiquitination assays, UMSCC22A cells were transfected as required. After 64 h, the cells were treated with 100 nM Bortezomib (MedChemExpress) for 8 h. Cells were washed with PBS and lysed in lysis buffer (1% Triton X-100, 0.5% deoxycholate, 1% NP-40, 50 mM Tris, 100 mM NaCl, 2 mM MgCl2, 10% glycerol) supplemented with 100 μM N-ethylmaleimide (Sigma) and protease inhibitor cocktail (Thermo Scientific). The lysates were centrifuged and incubated with 2 μg IkBα antibody (L35A5; 4814, CST) at 4 °C overnight with continuous rotation. A/G agarose beads were then added, and the lysate/antibody/bead mix was incubated at 4 °C overnight with continuous rotation. Beads were then washed in lysis buffer and boiled in Laemmli loading buffer prior to SDS PAGE and western blot analysis using an anti-HA antibody (C29F4; 3724, CST).
 **Immunofluorescent analysis by confocal microscopy**
Cells were seeded onto coverslips to adhere overnight. Cells were treated as required. At the required time, cells were fixed with 4% paraformaldehyde for 10 min and then permeabilised with 0.1% (v/v) Triton for 15 minutes. Cells were blocked in PBS containing 5% BSA for 1 hours. Cells were then incubated in primary antibodies in PBS with 1% BSA overnight at 4°C. Primary antibodies were used at a concentration of 1:400 (USP14 (D8Q6S; 11931, CST), RELA (8242, CST) and γH2AX (9718, CST). Cells were washed thoroughly in PBS and then incubated with Alex-fluor conjugated secondary antibodies 594 or Alexa 488 (1:1000; Invitrogen) in PBS with 4% BSA for 2 hours. DAPI was used to visualise nuclei. Coverslips were mounted onto slides with Prolong Gold (Invitrogen). Quantification of nuclear localisation was quantified from at 15 cells from three replicates were analysed as described (Ref).

**qRT-PCR analysis**
Cells were treated as indicated in the figure legends. Total RNA was isolated using Trizol and RNeasy Mini Kit (Qiagen) combined method per manufacturer’s protocol. cDNAs were synthesized using the High-Capacity cDNA Reverse Transcription Kit (Life Technologies) and qRT-PCR was performed on a QuantStudio 6 Flex Real-Time PCR system (Applied biosystems/Thermo Fisher Scientific). Predesigned Taqman primer/probe set were purchased from Life Technologies. Relative gene expression was normalized to GAPDH (Thermo Fisher Scientific, Cat#:4331182: GAPDH – hs99999905_m1) as an internal control, and fold changes were adjusted to the control samples. The cells in each experiment were transfected in duplicates, and each sample was assayed by qRT-PCR in triplicates. 2-ΔΔCt was calculated and used as an indication of the relative expression levels. Data were presented as mean ± standard deviation (SD) from triplicates, and statistical analyses were performed using the two-tailed, unpaired Student t-test.

**XTT viability assay**
Cells were treated as indicated in the figure legends. After 48 hours, XTT reagent was added per the manufacturer’s instructions and plates were read at 450 nm on a plate reader. Each condition was performed in triplicate.  **Colony formation assays**
Cells were treated as indicated in the figure legends. For colony formation assays, at the end of treatment, cells were trypsinized and reseeded in a six-well plate at 500 cells per well and left to form colonies for 10-14 days. Colonies were then stained (1% crystal violet, 25% methanol) and were counted manually. Each condition was performed in triplicate. For clonogenic survival assays, at the end of treatment, cells were trypsinized and reseeded in 12-well plates at 100-15000 cells per well (depending on radiation dose) and left to form colonies for 10-14 days. Colonies were then stained (1% crystal violet, 25% methanol) and were counted manually. Each condition was performed in triplicate. Radiation dose modifying factors (DMFs) were determined at 10% survival levels by dividing the radiation dose for control by the radiation dose for drug treated. DMFs > 1.0 indicate enhancement of radiosensitivity.

**Flow cytometry assays**
For cell cycle analysis, cells were treated as indicated in the figure legends. At each time point, cells were then harvested by trypsinization, collected by centrifugation and processed by following the protocol provided by Cycletest Plus DNA Reagent Kit (BD Biosciences). Analysis was performed using a FACS Canto flow cytometer (BD Biosciences). Data from 10,000 cells per sample analyzed using Flow-Jo analysis software (Tree Star). Each condition was performed in triplicate. For γH2AX analysis , cells were treated as indicated in the figure legends. At each time point, cells were tryspsinised, collected by centrifugation and fixed in 4% paraformaldehyde. Cells were then washed in 1% BSA in PBS and permeabilized in 90% methanol. Before the addition of antibodies, cells were blocked in 5% BSA in PBS. Cells we then incubated with a PE conjugated γH2AX antibody (1:100; 5763, CST) prior to analyzing on a FACS Canto flow cytometer (BD Biosciences). Gates were established using an anti-rabbit IgG negative control. Data from 10,000 cells per sample were analyzed using Flow-Jo analysis software (Tree Star).

**Annexin V assay**
Cells were treated as indicated in the figure legends. At each time point, cells were then harvested by trypsinization and collected by centrifugation. For HPV- cell lines, 1 × 10^6^ cells were then incubated in 100 μL Annexin V staining solution (TACS Annexin V kit; 4830-250-K; 10 μL 10× binding buffer, 10 μL propidium iodide, 1 μL Annexin V-FITC and 79 μL ddH2O) for 15 min at room temperature in the dark. Samples were diluted in 1× binding buffer before analysis by flow cytometry on a FACS Canto flow cytometer (BD Biosciences). For HPV+ cell lines, cells were washed in PBS and were then incubated with an Alexa Fluor^®^ 488 conjugated phosphatidylserine (PS) antibody (1:100; 16-256, Sigma-Aldrich). Cells were then stained with propidium iodide (PI) for 30 mins prior to being fixed in 4% paraformaldehyde. Cells were then analyzed on a FACS Canto flow cytometer (BD Biosciences). Data from 10,000 cells per sample analyzed using Flow-Jo analysis software (Tree Star). Early apoptotic cells were defined as Annexin positive/PI negative and late apoptotic cells was defined as Annexin positive/PI positive. Each condition was performed in triplicate.
