# Supplementary material for: Inhibition of USP14 promotes TNFα-induced cell death in head and neck squamous cell carcinoma (HNSCC)
Source: Cell Death Differ. 2023 Apr 13;30(5):1382–96. doi: 10.1038/s41418-023-01144-x (PMC10154301; doi:10.1038/s41418-023-01144-x)
Supplement: Supplementary file 5 — Uncropped Blots [file 41418_2023_1144_MOESM5_ESM.pdf]

Figure 2

2A)

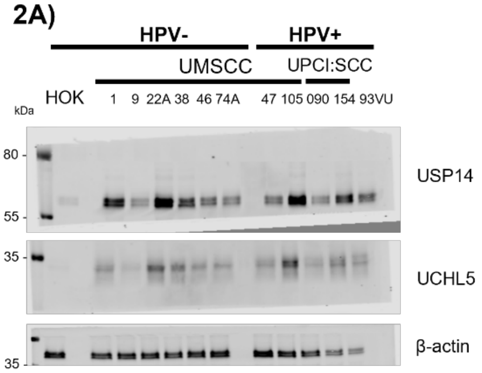

Figure 3

3G)

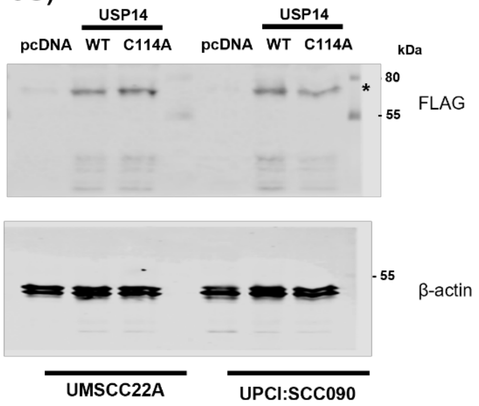

Supplementary Figure 2

Supp 2A)

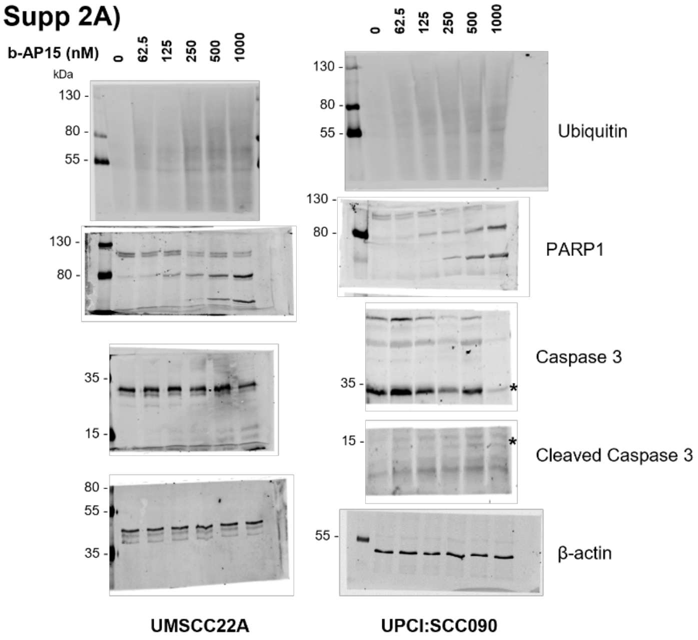

Figure 3

3A)

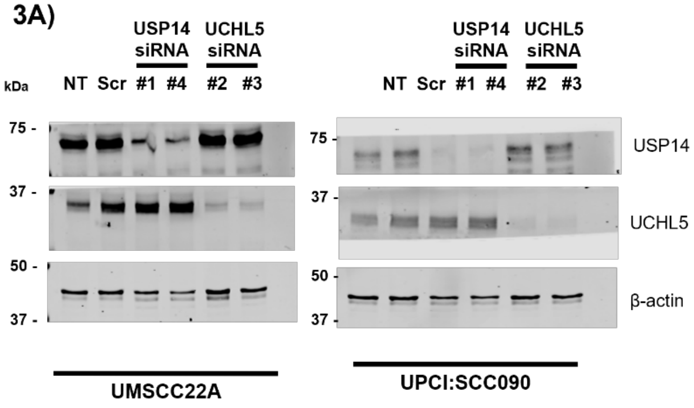

Figure 4

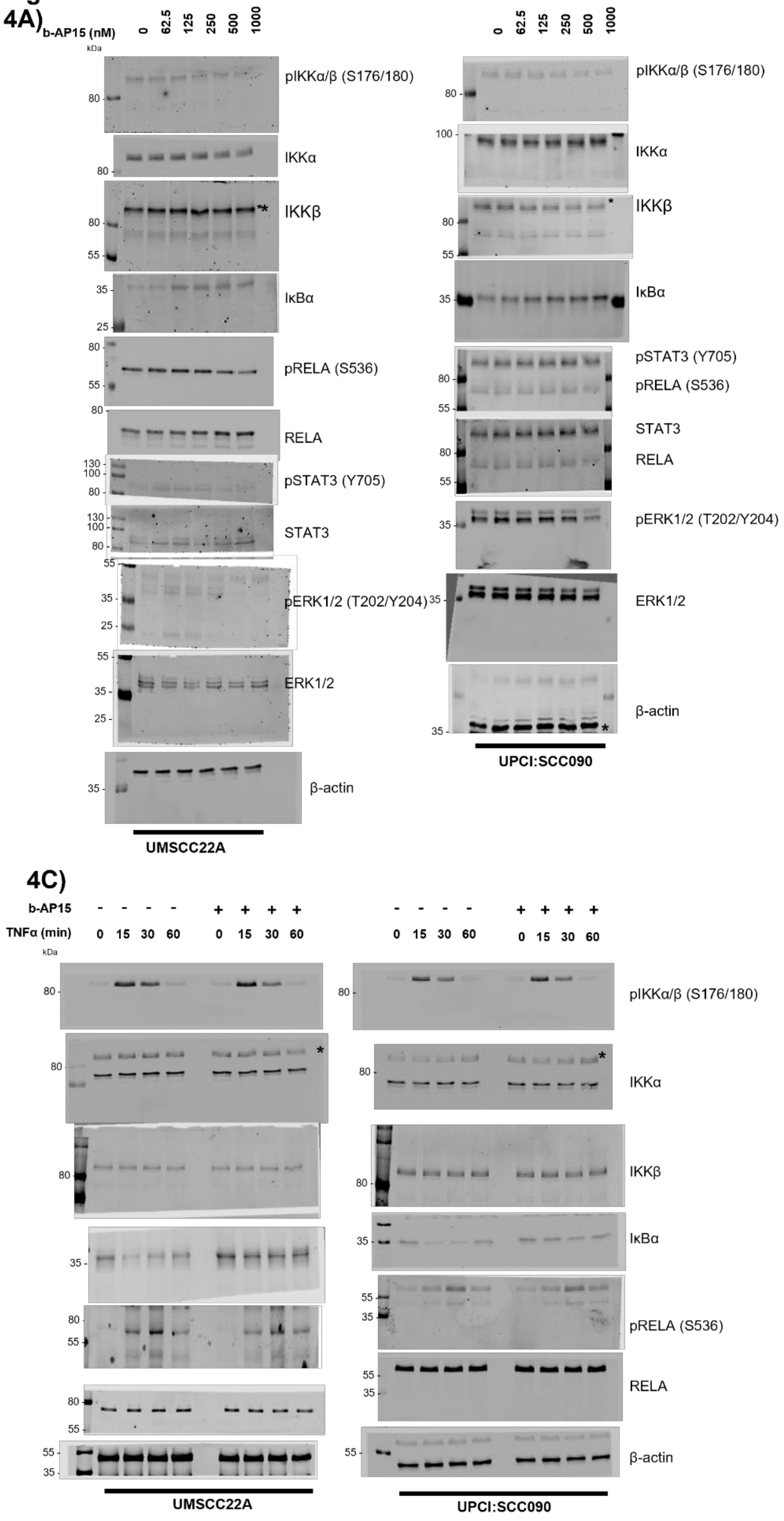

4A)
b-AP15 (nM)
0 62.5 125 250 500 1000
kDa
pIKKα/β (S176/180)
IKKα
IKKβ
IκBα
pRELA (S536)
RELA
pSTAT3 (Y705)
STAT3
pERK1/2 (T202/Y204)
ERK1/2
β-actin
UMSCC22A
UPCI:SCC090
4C)
b-AP15
TNFα (min)
0 15 30 60 0 15 30 60
pIKKα/β (S176/180)
IKKα
IKKβ
IκBα
pRELA (S536)
RELA
β-actin
UMSCC22A
UPCI:SCC090

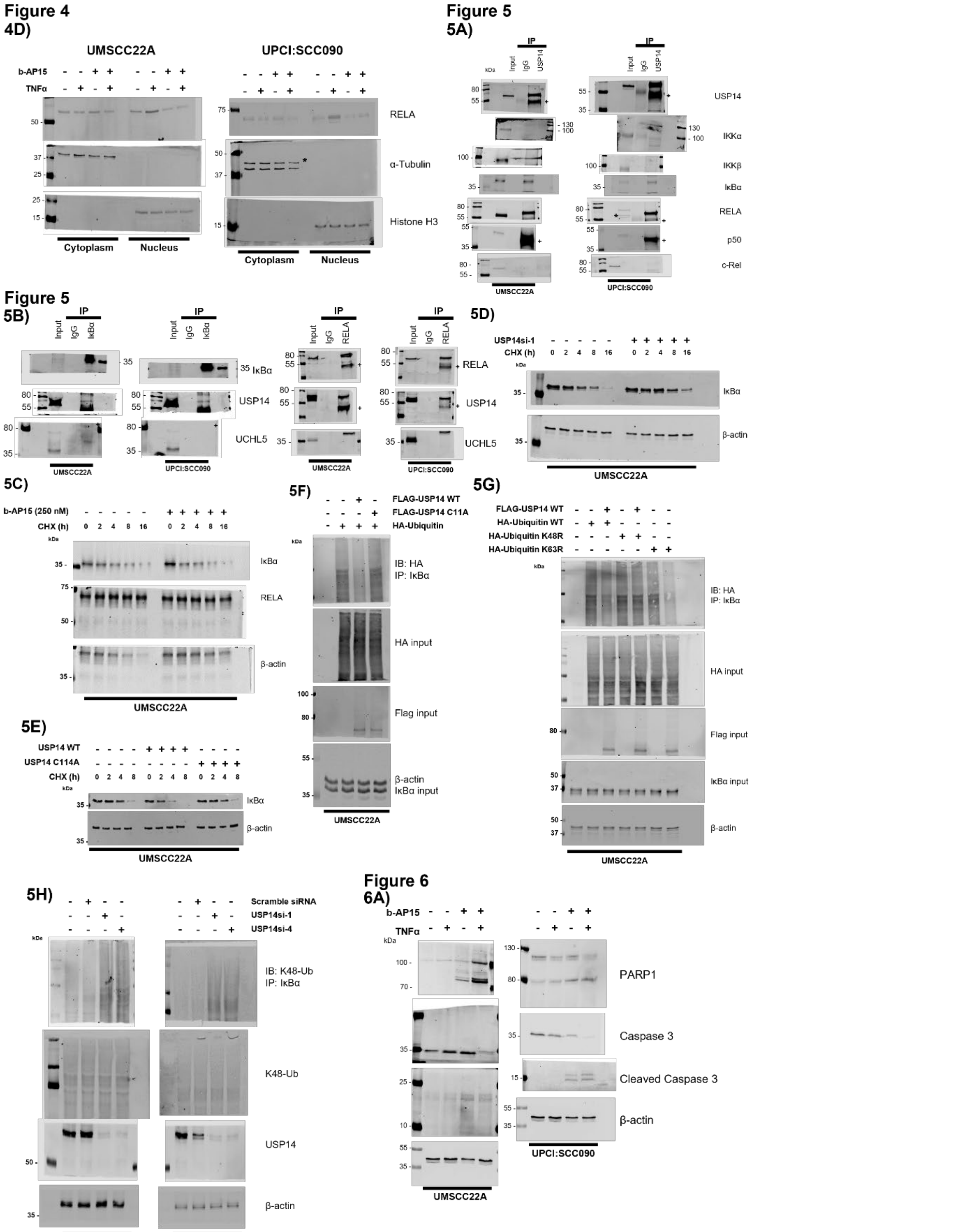

Uncropped blots. * indicates the correct band where there are multiple bands. + indicated IgG bands from immunoprecipitation experiments.
