# Supplementary material for: Inhibition of USP14 promotes TNFα-induced cell death in head and neck squamous cell carcinoma (HNSCC)
Source: Cell Death Differ. 2023 Apr 13;30(5):1382–96. doi: 10.1038/s41418-023-01144-x (PMC10154301; doi:10.1038/s41418-023-01144-x)
Supplement: Supplementary file 6 — Author Contribution List [file 41418_2023_1144_MOESM6_ESM.pdf]

DECLARATION OF CONTRIBUTIONS TO ARTICLE

ADMC

Manuscript Number:	Journal Name:	
	Cell Death & Differentiation	(the 'Journal')

Proposed Title of the Contribution:

Inhibition of USP14 promotes TNF-induced cell death in Head and Neck Squamous Cell Carcinoma (HNSCC) (the 'Contribution)

Author(s):

Ethan L. Morgan, Tiffany Toni, Ramya Viswanathan, Yvette Robbins, Xinping Yang, Hui Cheng, Sreenivasulu Gunti4, Angel Huynh, Anastasia L. Sowers, James B. Mitchell, Clint T. Allen, Zhong Chen and Carter Van Waes (the 'Authors')

For all *CDD* articles, each person named as an author in the published version must be able to show he or she has contributed substantially to the article.

Authorship credit should be based on 1) substantial contributions to conception and design, acquisition of data, or analysis and interpretation of data; 2) drafting the article or revising it critically for important intellectual content; and 3) final approval of the version to be published. Authors should meet conditions 1, 2 and 3.

Any person who cannot be shown to have made a substantial contribution to the article cannot be listed as an author in the final version. The name of any person who is deemed to have made a minor contribution can, however, appear in the Acknowledgments section of the article.

Please complete the table below to indicate the contributions of all named authors to the manuscript.

Author Full Name:	Specification of Contribution to the Manuscript:
Ethan L Morgan	Designed all experiments, performed the majority of experiments, analysed and interpreted all data, supervised the project and wrote the initial manuscript
Tiffany Toni	Performed several experiments and data analysis, contributed to manuscript preparation and approved the final manuscript
Ramya Viswanathan	Performed several experiments and data analysis, contributed to manuscript preparation and approved the final manuscript
Yvette Robbins	Performed several experiments and data analysis, contributed to manuscript preparation and approved the final manuscript
Xinping Yang	Performed several experiments and data analysis, contributed to manuscript preparation and approved the final manuscript
Hui Cheng	Performed several data analyses, contributed to manuscript preparation and approved the final manuscript
Sreenivasulu Gunti	Created reagents for the use in the manuscript, contributed to manuscript preparation and approved the final manuscript
Angel Huynh	Provided reagents and technical support for the project, contributed to manuscript preparation and approved the final manuscript
Anastasia L Sowers	Provided equipment and technical support for the project, contributed to manuscript preparation and approved the final manuscript
James B Mitchell	Provided equipment and technical support for the project, contributed to manuscript preparation and approved the final manuscript
Clint T Allen	Assisted data analysis, provided funding, supervised the project, contributed to manuscript preparation and approved the final manuscript
Zhong Chen	Assisted with experimental designed and analysis, supervised the project, contributed to manuscript preparation and approved the final manuscript
Carter Van Waes	Designed and analysed data, supervised the project, provided funding and wrote the initial manuscript

Please complete the table below to indicate the contributions of all named authors to the figures.

Figure 1:
ELM, HC, ZC and CVW designed experiment. ELM and HC performed experiments and analysed data.

Figure 2:
ELM, ZC and CVW designed experiment. SG and AH provided reagents. ELM performed experiments and analysed data.

Figure 3:
ELM, ZC and CVW designed experiment. ELM performed experiments and analysed data.

Figure 4:
ELM, RV, XY, ZC and CVW designed experiment. ELM, RV and XY performed experiments and analysed data.

Figure 5:
ELM, ZC and CVW designed experiment. ELM performed experiments and analysed data.

Figure 6:
ELM, ZC and CVW designed experiment. ELM performed experiments and analysed data.

Signed for and on behalf of the Author(s):	Print Name:	Date:

Please complete the table below to indicate the contributions of all named authors to the figures.

Figure 1:

Figure 7.

ELM, TT, ALS, JBM, ZC and CVW designed experiment. ELM performed experiments and ELM, ALS and JBM analysed data.

Figure 2:

Figure 8.

ELM, TT, YR, AH, CTA, ZC and CVW designed experiment. ELM, TT, RV, YR and AH performed experiments and analysed data.

Figure 3:

Figure 4:

Figure 5:

Figure 6:

Signed for and on behalf of the Author(s):	Print Name:	Date:
[signature]	ETHAN L MORGAN	08/19/2022
